# Are children really more creative than adults? A mixed-methods examination of creative performance and experience

**DOI:** 10.1371/journal.pone.0346553

**Published:** 2026-10-21

**Authors:** Janika Saretzki, Ines Fratzl, Baptiste Barbot, Mathias Benedek

**Affiliations:** 1 Department of Psychology, University of Graz, Graz, Austria; 2 Department of Psychology, LMU Munich, Munich, Germany; 3 Psychological Sciences Research Institute, UCLouvain, Louvain-la Neuve, Belgium; 4 Child Study Center, Yale University, New Haven, Connecticut, United States of America; A'Sharqiyah University, OMAN

## Abstract

It is widely believed that children are more creative than adults, yet direct empirical tests of this assumption are scarce. A qualitative pre-study indicated that creativity is commonly attributed to preschoolers and associated with intrinsically motivated engagement, informing the design of the main study. 92 preschool children and 70 adults completed a divergent thinking task and two drawing tasks varying in structure (free vs. goal-directed). Independent raters evaluated all products for novelty, effectiveness, and overall creativity; participants reported motivation, enjoyment, confidence, and anxiety. Adults outperformed children on externally rated creativity, particularly in structured tasks. In contrast, children reported higher confidence and motivation, lower anxiety, and—except in the structured drawing task—greater enjoyment. Together, these findings challenge the view that children are generally more creative than adults by showing that their apparent advantage lies in confident and intrinsically motivated engagement, whereas creative thinking performance increases with age.

## Introduction

Children are widely believed to be more creative than adults—a conviction that permeates public discourse, educational practice, and everyday intuition [1–3]. At the same time, this belief appears to contrast with evidence from age-normed creativity tests and meta-analytical work, which typically show a steady increase in creative thinking performance from childhood into adulthood [4–7]. Surprisingly, direct empirical comparisons of children’s and adults’ creativity remain scarce, leaving open the possibility that children may excel in other, less examined facets of creativity beyond conventional test performance. Therefore, the present research revisits the popular notion of children’s superior creativity by adopting a broader, multidimensional perspective.

International survey data indicate that a majority of people endorse the statement that children are more creative than adults (68–77%; [1,2]). Similar views are commonly held among educators such as teachers in the US and Japan (63–68%; [3,8]). These views align with another popular conception stating that schools have a detrimental impact on children’s creativity [1,9], a perspective widely disseminated through influential TED talks and books [10,11].

In contrast to the pervasiveness of these beliefs, the most systematic empirical evidence on age differences in creativity comes from age-normed tests of divergent thinking (DT), which reflect established indicators of creative thinking ability. Across widely used instruments such as the Torrance Tests of Creative Thinking [4], the Evaluation of Creative Potential (EPoC) [5], the Test for Creative Thinking – Drawing Production [6], and the Test of Creative Imagery Abilities (TCIA) [12], DT performance typically increases from early childhood through adolescence, with relatively stable levels in adulthood [7,13,14]. Notably, evidence further suggests that school education does not undermine but rather facilitates creativity [15].

The apparent discrepancy between popular beliefs and empirical findings raises the question of whether perceived differences in creativity between children and adults are obscured by how creativity is conceptualized and assessed. Creativity is a multifaceted construct that extends beyond creative thinking ability to include relevant traits, skills, and behavioral components. Specifically, creative behavior and achievement is known to develop and arise from the interplay of cognitive abilities (e.g., DT ability), core personality traits (e.g., openness to new experiences), creative self-beliefs (e.g., creative confidence) and expertise in the context of environmental influences [16,17]. Some of these facets, such as domain-specific expertise and lifetime creative achievement, depend on accumulated knowledge and prolonged practice and therefore typically increase with age, favoring adults [18]. For example, professional or eminent levels of creativity (i.e., Pro-c and Big-C) are typically observed only at later career stages or even assigned posthumous [19,20]. Other facets may align more closely with popular intuitions about children’s creativity. Children may engage with creative tasks in a more play-like manner [21,22] reflecting high openness [23], and intrinsic motivation [24], thus enjoying the task without being overly concerned about the outcome [25]. Adults, in contrast, are more aware of their strengths and weaknesses [26] and typically more sensitive to external expectations and social evaluation [27], which may support goal-directed performance via metacognition and self-regulation [28,29] but also increase worries and constrain risk-taking [30,31]. Considering both cognitive and non-cognitive aspects of creativity therefore appears essential when examining whether children are more or less creative than adults.

### The present study

The present research revisits the assumption that children are more creative than adults by adopting a broader, multidimensional perspective. In a qualitative pre-study, we examined laypeople’s assumptions about how and at which age children are thought to be more creative than adults. Guided by these insights, the main quantitative study compared preschool children and adults across multiple measures of creativity including more play-like and test-like task conditions, scored for novelty, effectiveness, and overall creativity. In addition to creative performance tasks, we assessed emotional experience and confidence during tasks to capture non-cognitive dimensions of creativity.

Based on prior research on general developmental trends in DT performance [e.g., 7], we expected adults to show higher creative performance than preschool children. Given the limited evidence on age differences across non-cognitive creativity measures, group comparisons for these measures were mainly exploratory.

## Materials and methods

The present study employed a sequential mixed-methods design, consisting of a qualitative pre-study followed by a quantitative main study. The qualitative component examined lay conceptions of creativity in children and adults and informed the design and interpretation of the subsequent quantitative study. The study protocol was approved by the local ethics committee (GZ 39/119/63 ex 2020/21).

All materials, data, and analysis scripts, and supplemental analyses related to the current study are publicly accessible in an OSF repository: https://osf.io/wbaj6.

### Qualitative pre-study

#### Participants and procedure.

10 adult laypeople (60% female) with a mean age of 29.70 years (*SD* = 2.36) participated in individual semi-structured interviews designed to elicit beliefs about creativity in children and adults after giving verbal informed consent. Additional information on participant characteristics and study materials is available in the OSF repository. Participants were recruited via convenience sampling from the local community and the researchers’ personal networks. Interviews addressed perceived developmental changes in creativity (including the age at which children are considered the most creative) and beliefs about characteristics and behaviors associated with creativity in children versus adults. Interviews were audio-recorded, transcribed verbatim, and analyzed using qualitative content analysis. The sample size was consistent with recommendations for achieving thematic saturation in interview-based research [32,33] and appropriate for the exploratory aim of the pre-study.

### Coding and analysis

Interview transcripts were analyzed following Mayring’s (2014) approach to qualitative content analysis [34]. Coding categories were developed inductively and refined with reference to prior research on implicit theories of creativity (the resulting coding scheme is provided in the supplemental material of the qualitative study). The coding scheme captured personal characteristics (e.g., originality, openness to experience), behavior manifestations of creativity (e.g., concrete expressive behavior, domain specificity), and perceived environmental or contextual conditions (e.g., freedom from external constraints).

Two trained raters independently coded all transcripts. Although interview questions explicitly targeted either children or adults, participants frequently referred to both groups within a single response. Statements were therefore coded independently of the interview question and categorized according to whether they referred to creativity in children, adults, or both. Interrater reliability was assessed at the statement level using Jaccard similarity, which indicated moderate agreement (*M* = .596, 95% CI [.495,  .687]). Discrepancies were subsequently resolved through consensus coding. A descriptive overview of the resulting category distributions for children and adults is shown in Fig 1.

**Fig 1:**
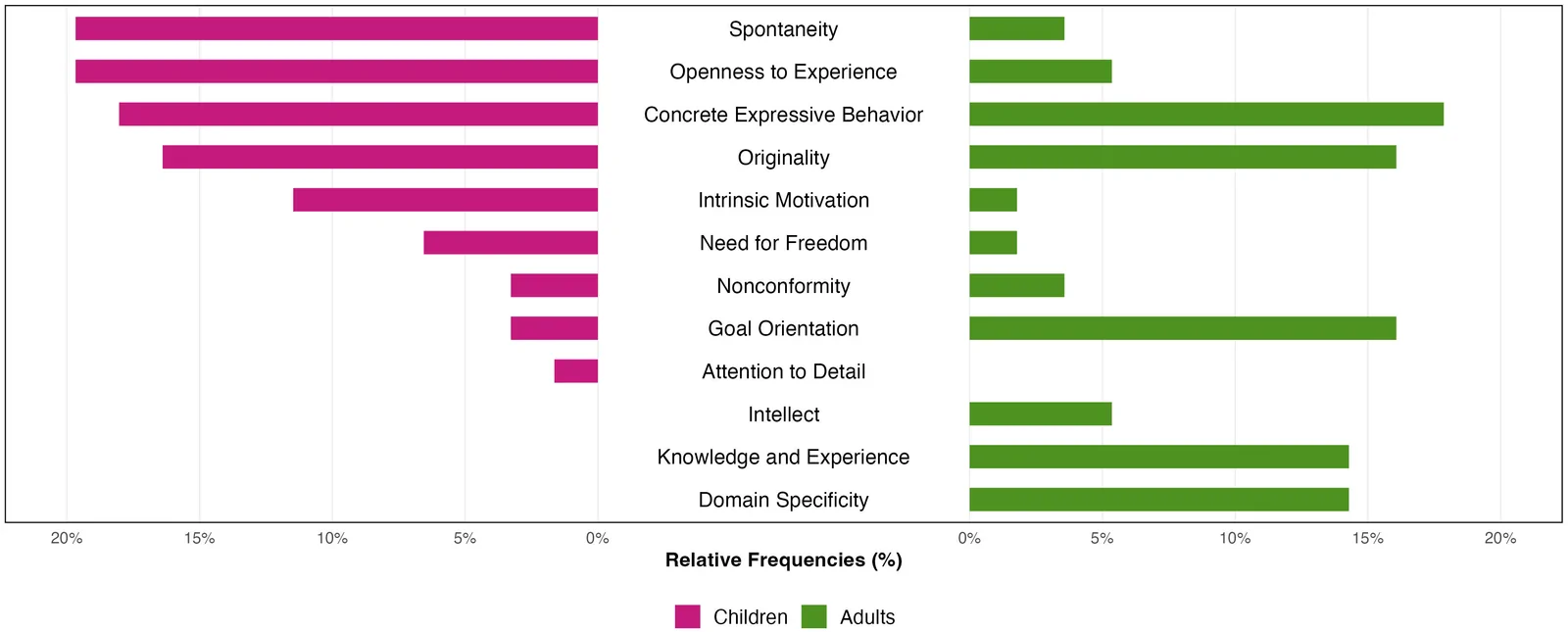
Differences in lay descriptions of creativity in children and adults. Bars represent the relative frequencies of category assignments for child- and adult-related statements, sorted by children category frequency.

### Design implications for the main study

Qualitative findings suggested that children’s creativity is commonly associated with openness, spontaneity, intrinsic motivation, and freedom from external constraints, whereas adults’ creativity was primarily associated with goal orientation, accumulated knowledge and experience, and domain specificity. Concrete expressive behaviors (e.g., drawing, storytelling, experimenting) were mentioned for both groups but contextualized differently: children’s creativity was typically described in terms of playful exploration, spontaneous experimentation, and low inhibitions, whereas adults’ creativity was more often characterized as more product- and goal-oriented, and situated within specific domains such as profession or hobbies. These insights motivated us to examine creative performance under two conditions that might favor children versus adults [21, but see 35], respectively: 1) an unconstrained, play-like condition that does pose a clear goal or time-limit (cf. free drawing task), and 2) a constrained, test-like condition that has a clear goal and time-limit (cf. structured drawing task). Additionally, we included the well-established alternative uses task for reference with the rich literature using this task [36] and scored creative performance independently for novelty, effectiveness and overall creativity [37], as children’s playful exploration behavior might yield more novel yet less effective responses.

Environmental references centered on freedom, with children frequently described as having fewer external constraints and greater latitude for exploration, while adults were portrayed as facing time pressure, social expectations, and performance demands. Therefore, we decided to measure subjective experience during the creative tasks besides actual task performance. Finally, since participants predominantly located peak creativity in early childhood, particularly shortly before school entry, we decided to focus on preschool children in the comparison with adults.

### Quantitative main study

#### Participants.

The sample comprised 92 preschool children (52.2% female; *M* = 5.67 years, *SD* = 0.46; range: 4.66–7.26) and 70 adults (67.1% female, 1.4% diverse; *M* = 25.31 years, *SD* = 5.90; range: 20.04–52.10; 85% were between 20 and 30 years). Children were recruited from nine local kindergartens in their final year prior to school entry. Adults were recruited from the general population. The study aimed for largely representative samples within preschoolers and adults and therefore did not apply other formal inclusion/exclusion criteria. Written informed consent was obtained from all adult participants and from children’s legal guardians.

#### Tasks and measures.

All participants completed three creativity tasks in a fixed order—an alternate uses task (AUT) [38], a widely used divergent thinking paradigm [see also 36], a free drawing task, and a structured drawing task—followed by self-report questions. The procedure was initially pilot-tested with two children (four and seven years old) to evaluate the comprehensibility and feasibility of the tasks and instructions across different developmental levels; the results informed final task adjustments.

In the AUT, participants generated as many creative uses as possible for a *tin can* within 3 minutes. To account for the children’s limited experience with the object, an actual tin can was provided to all participants (children and adults), allowing them to explore it during the task. Responses were given orally, recorded, and later transcribed, consistent with established procedures for assessing creativity in young children [5].

Then, a creative drawing task was completed under unconstrained and constrained conditions (i.e., free vs. structured drawing; representative examples are provided in the OSF supplementary materials): In the free drawing task, participants were given an A4 paper and a variety of colored pens and invited to draw freely what they wanted. This task was not framed as test but rather as a familiarization with the test materials for a subsequent task to ensure a play-like condition. No explicit time limit was imposed or mentioned, but participants were asked to eventually conclude the drawing after about 8 minutes. In the structured drawing task, participants were instructed to draw a fantasy animal that does not exist, using the same materials as in the free drawing task. The task duration was set and communicated to a maximum of 8 minutes. Task completion time was recorded in all three creativity tasks.

After completing all tasks, participants reported their task-related enjoyment (“How much fun was the task?”), motivation (“How much did you like doing this task?”), self-efficacy (“How certain were you to perform well before the task?”), self-appraisal (“How well do you think you performed the task?”), and anxiety (“How much were you worried/anxious to not perform well on the task?”) in each task with single self-report items. Following similar assessments with young children, we used brief items that were communicated orally in an age-appropriate language using a five-point scale anchored with emoticons [e.g., 39]. For each item, all response options were described by the experimenter, and the question was rephrased if necessary. Participants also estimated the frequency of their creative engagement during leisure time on a four-point scale ranging from “nearly every day” to “at least once per month“ (for children, this information was provided by their parents), provided a global self-assessment of their creativity on a five-point scale ranging from 1 (low) to 5 (high), and indicated whether they considered children or adults to be more creative. The exact wording of all items and response scales is available in the OSF repository (see Materials).

#### Scoring of creative performance.

All creative products were evaluated by five experienced raters blind to participants’ age group and task condition. The AUT resulted in a total of 670 responses that were pooled and presented in alphabetic order for the rating. The two drawing tasks resulted in 324 drawings (162 participants x two drawing conditions) which were presented in a randomized order for the rating. The quality of each AUT response and drawing was rated on five-point Likert scales for novelty, effectiveness, and overall creativity.

Raters received initial training that involved familiarization with the rating scales and criteria, followed by supervised practice on a subset of responses/drawings; after discussing uncertainties, the remaining responses were subsequently coded independently.

Interrater reliability was high across tasks and dimensions, with Cronbach’s α ranging from  .789 to  .980. Full reliability coefficients, including 95% confidence intervals, are reported in the Supplemental Analyses (see S1 Table). Response-level correlations among novelty, effectiveness, and overall creativity ratings replicated that creativity evaluations are more strongly driven by the perceived novelty than effectiveness; detailed analyses are reported in the Supplemental Analyses (see S2 Table).

Ratings were averaged across raters. For the AUT, a max-3 aggregation approach was applied to reduce response fluency confounds by focusing on a fixed number of responses; specifically, scores reflect the average ratings of the three highest-rated responses per participant [40].

### Procedure

All participants were tested individually between August 1, 2021, and April 30, 2022, by one of the authors, who was not familiar with the participants prior to testing. The procedure was identical across both groups, with minor age-appropriate adaptations for children (e.g., simplified language and additional comprehension checks). After explaining the procedure and obtaining written informed consent, the experimenter administered the creativity tasks in a fixed order: demographic questions, the AUT, the unstructured drawing task, the structured drawing task, self-assessments for all three tasks, and final creativity reports. The total study duration was approximately 30 min.

### Statistical analyses

All analyses were conducted using R (version 4.5.1). Primary analyses examined group differences between children and adults using independent-samples *t*-tests for continuous outcomes chi-square tests for categorical variables. Effect sizes are reported as Cohen’s *d*, *r*, or Cramér’s *V*, as appropriate. To examine whether group differences varied systematically across task formats, 2 (group: children vs. adults) x 3 (task: AUT, free drawing, structured drawing) mixed analyses of variance (ANOVAs) were conducted, with partial eta squared (η_p_^*2*^) reported as effect size (see Supplemental Analyses, S3 Table).

All tests were two-tailed. To account for multiple comparisons, a conservative significance threshold of *p* < .001 was applied. This threshold approximated the level of stringency achieved by common correction procedures and ensures a consistent criterion across analyses.

## Results

### General creative engagement, creative self-concept, and beliefs about creativity

Before turning to task-based measures, we examined group differences in creative leisure activities, creative self-concept, and beliefs about creativity (see Fig 2).

**Fig 2:**
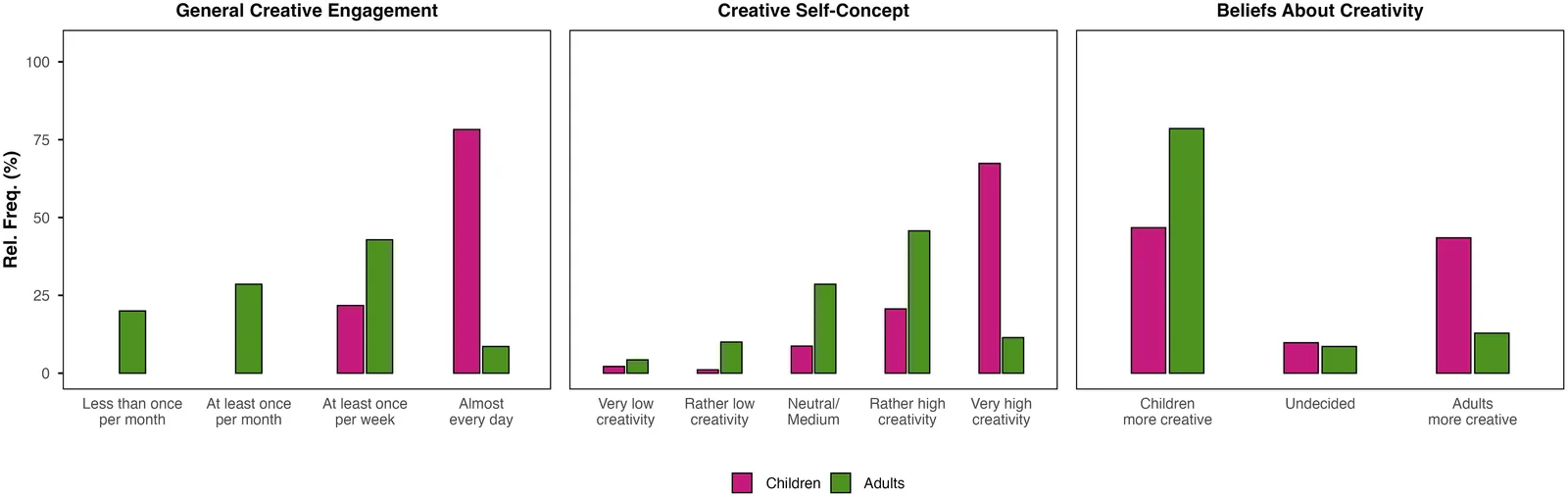
General creative engagement, creative self-concept, and beliefs about creativity. For preschool children, ratings of general creative engagement were provided by parents, whereas all other measures were self-reported.

Preschool children engage in creative activities more frequently (*M* = 3.78, *SD* = 0.41) than adults (*M* = 2.40, *SD* = 0.91; Wilcoxon rank-sum test, *W* = 5,804, *p* < .001; *r* = .742, 95% CI [.660,  .815]), most commonly indicating engagement *almost every day*, whereas adults most frequently reported engaging *at least once per week*. Children (*M* = 4.50, *SD* = 0.87) also reported a higher creative self-concept than adults (*M* = 3.50, *SD* = 0.97; Wilcoxon rank-sum test, *W* = 5,136, *p* < .001; *r* = .541, 95% CI [.421,  .666]): Whereas the majority of children endorsed *very high creativity*, adults most commonly reported a *rather high* creative self-concept.

When asked directly whether children or adults are more creative, response distributions differed between groups (*χ*²(2) = 19.05, *p* < .001; Cramér’s *V* = 0.343, 95% CI [0.217, 0.482]). Children’s responses were relatively balanced (46.7% children, 43.5% adults, 9.8% both/no clear decision), whereas a clear majority of adults (78.6%) indicated that children are more creative (12.9% adults, 8.6% both/no clear decision).

### Task-based creative performance, task duration, and subjective experience

Fig 3 summarizes group differences across tasks for externally rated creative performance (novelty, effectiveness, creativity), and self-reported subjective experience (self-efficacy, motivation, enjoyment, self-appraisal, and anxiety). All inferential tests and individual group comparisons are reported in the Supporting Information (see S3 and S4 Tables).

**Fig 3:**
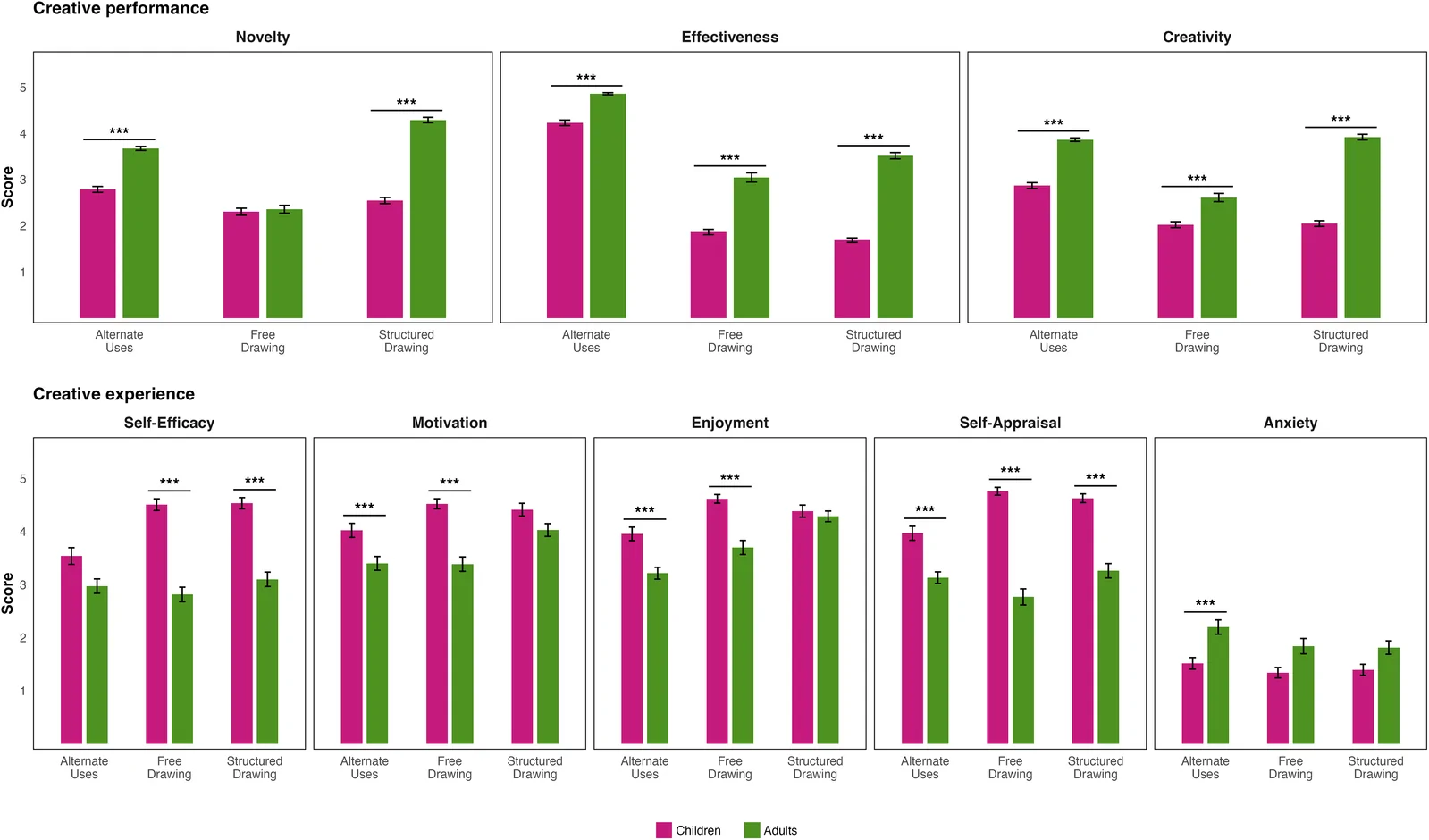
Creative task performance and subjective experience in the three creativity tasks for children and adults. Asterisks indicate statistical significance of between-group comparisons with t-tests. *** *p* < .001.

Adults showed consistently higher task performance than the preschool children in the AUT and the structured drawing task, as their ideas and drawings were rated as more novel, effective, and creative. In the free drawing task, adults also produced more effective and creative drawings than children, but groups did not differ in terms of novelty as adults often chose similarly common drawing topics. This pattern is consistent with the task instructions, as only the structured drawing task explicitly encouraged the production of a creative response. Significant interactions for all three performance measures indicated that smaller group differences were observed for novelty and creativity in the unstructured drawing, whereas group differences in effectiveness were highest in the structured drawing task.

Interestingly, children spent less time than adults on the timed AUT [*M* = 1.92, *SD* = 0.87 vs. *M* = 2.72, *SD* = 0.61; *t*(159) = −6.46, *p* < .001] and structured drawing task [*M* = 2.71, *SD* = 2.32 vs. *M* = 6.58, *SD* = 1.92; *t*(159) = −11.63, *p* < .001]; in *t*he unstructured drawing task, which was untimed, children (*M* = 5.06, *SD* = 4.66) and adults (*M* = 4.16, *SD* = 2.90) spent a similar amount of time [*t*(154) = 1.50, *p* = .134]. All group comparisons were additionally examined while con*t*rolling for time on task, which did not change the overall pattern of results (see Supplemental Analyses, S3 Table).

Children reported a more positive subjective experience during the creative performances. They indicated higher motivation in the AUT and the free drawing task, and higher self-efficacy in both drawing tasks. Across all three tasks, children reported higher self-appraisal. Moreover, children experienced less anxiety in the AUT and enjoyed both the AUT and the free drawing more than adults, but groups equally enjoyed the structured drawing task. Significant interactions for motivation and enjoyment indicated that adults generally felt more comfortable in the structured drawing task, whereas children enjoyed the free drawing task more.

Together, adults showed overall more creative performance, with the strongest effects in the goal-directed tasks (AUT and structured drawing task), whereas children reported a more positive creative experience, particularly in the free drawing task.

## Discussion

The present study examined the popular belief that children are more creative than adults. Informed by a qualitative pre-study, we compared preschool children’s and adults’ creative performance and subjective experience across three creative tasks. Adults outperformed preschool children by producing more novel, effective, and creative ideas, particularly in tasks with explicit goals and evaluative constraints. Children, in contrast, showed higher creative confidence and engage more often in creative activities. These findings suggest a clear dissociation: Adults show higher creative performance, whereas children enjoy creative tasks more and engage in them more often.

### Are children considered more creative than adults?

Consistent with previous work, adults viewed children as more creative. While 63–77% endorsed this view across cultures [e.g., 1], endorsement was slightly higher here (80% in the pre-study and 78% in the main study). Extending prior evidence, we also asked children whom they perceive as more creative. Children showed no similarly strong preference, with roughly equal proportions attributing greater creativity to children versus adults. These findings suggest that the belief in children’s superior creativity is robust among adults, whereas children’s own judgments appear less polarized.

### Are children more creative than adults?

When creativity is operationalized as creative (cognitive) potential, adults outperformed preschool children in generating ideas in verbal and figural tasks. This pattern is consistent with developmental research and test norms, which show age-related increases in creative performance across childhood and adolescence [4,6,7,13,41]. By evaluating products not only holistically but also separately in terms of novelty and effectiveness [42], we ruled out the possibility that children generate more unique but less effective ideas. Adults’ output was more novel and effective in tasks requiring creativity; only in the free drawing task did both groups produce similarly common drawings. These age differences likely reflect cognitive and metacognitive control in creative thinking [28,43], which are subject to neurocognitive development [44] supported by education [45,46]. Even within 5- to 6-year-olds, higher executive function already predicts higher creative performance under conditions that involve metacognitive regulation [47].

A different picture emerged for subjective experience and everyday engagement. While adults demonstrated higher creative ability in standardized tasks, children enjoyed the creativity tasks more, reported higher motivation and confidence, and showed lower anxiety. They also reported more frequent creative engagement and a higher creative self-concept. These findings suggest that the common perception of young children as “more creative” may stem less from their output quality and more from their affective-motivational experience and agentic action.

Following the *Creative Behavior as Agentic Action* model [48], creative behavior is not just a function of ability, but also of creative confidence beliefs. In our study, children exhibited lower creative thinking potential than adults, yet higher creative confidence and overall higher creative agency, in terms of their intrinsic willingness to engage in creative behavior. This heightened agency in children can also be interpreted through the lens of *Self-Determination Theory* and relevant motivational behaviors [49]. Children’s higher enjoyment and confidence could be seen as indicators of need-supportive contexts, where the basic psychological needs for autonomy and competence are satisfied, likely because children perceive creative tasks as low-stakes, playful activities [50]. Conversely, the adults’ higher anxiety suggests need-thwarting conditions—where evaluative pressure or rigid self-expectations may undermine their autonomy and competence.

Notably, children’s high confidence may partly reflect still-developing metacognitive abilities [51], and be reinforced by the inflated praise children often receive for their performance [52]. Yet, creative self-beliefs are key drivers of creative engagement and support the realization of creative potential [48], which may help explain why children are widely perceived as “more creative” despite showing lower creative performance in standardized tasks.

### The role of task constraints

Task format further differentiated performance and experience. The more constrained task conditions maximized performance differences, whereas the less constrained task conditions maximized experience differences. This pattern suggests that children’s creative engagement is sensitive to evaluative constraints [21]. In untimed, low-constraint contexts, children also tended to persist longer, consistent with time-on-task as an indicator of intrinsic motivation [24,53]. Together, these findings underscore intrinsic motivation as a key driver of children’s creative engagement, suggesting that their creative potential is particularly expressed in autonomy-supportive, low-evaluative contexts.

### Why do laypeople view children as more creative?

Given the observed dissociation between performance and experience, why do adults nevertheless view children as more creative? Judgments of creativity may be influenced by affective and behavioral cues, such as enthusiasm, confidence, and expressive engagement, which are salient in everyday contexts [54,55]. Children’s enjoyment and frequent engagement may therefore serve as cues for perceived creativity, whereas adults’ more restrained engagement and higher anxiety—especially under evaluation—may attenuate perceived creativity despite higher creative thinking potential. Thus, the public belief that children are “more creative” may reflect a broader, engagement-focused definition of creativity [56]. Analogously, a “sporty” person is often someone who regularly engages in sports rather than wins competitions. In sum, these findings help debunk, or at least add nuance to, the popular myth that young children are generally more creative than adults. Such simplistic views underestimate the effort involved in creative performance, similar to the belief that abstract art cannot be distinguished from children’s doodles [57].

### Limitations and future directions

Several limitations should be noted. First, cross-sectional comparisons are susceptible to cohort effects and do not enable causal inference. Second, comparing preschool children to adults is challenged by developmental differences in cognitive, language, and experience with test-like settings. Although procedures were adapted to be age-appropriate, especially the findings for self-report measures need to be interpreted cautiously given known response tendencies in children (e.g., more extreme responding; [58]) and the developing understanding of abstract constructs like “creativity” [46]. Third, the study focused on a narrow age range; future work should examine developmental trajectories and their relation to self- and conceptual knowledge about creativity [59]. Fourth, other creativity tasks (e.g., insight problem-solving) and more open-ended or collaborative forms of creative activity could be included, and task order should be counterbalanced to limit fatigue effects. Future research may also consider a broader range of evaluation criteria, such as narrative coherence and collaborative idea development when comparing creativity across age groups. Fifth, creative products were rated by adults; children might evaluate children’s products differently. In addition, we did not assess participants’ professional background or creative expertise, which may represent an additional source of variability in creative performance. Moreover, although raters were blind to participants’ age group, age-related characteristics of the drawings may have allowed group membership to be inferred in some cases. Finally, as the findings are based on samples drawn from a specific cultural context, future research could examine how lay beliefs and creative expression vary across different populations and backgrounds. Yet, another avenue for future work would be to confront adults viewing children as more creative with the findings of this study and ask them to justify or elaborate their views in the light of these results.

## Conclusion

The notion that children are generally more creative than adults is overly simplistic. Preschool children are less creative than adults in terms of their creative thinking potential, but they experience creative activity more positively, engage in it more frequently, and show higher creative confidence reflecting overall higher creative agency. It is likely that everyday judgments of children’s “higher creativity” are driven more by these salient engagement-related cues than by differences in creative output. Future research should test whether adults’ lower creative self-views reflect a true decline with age or more calibrated self-knowledge.

## Supporting information

S1 Table Interrater reliability [ICC(3, 5)] of scores across creativity tasks. Analyses of variance-based 95% CIs are reported in brackets.
(DOCX)

S2 Table Response-level correlations among novelty, effectiveness, and overall creativity ratings across creativity tasks. * *p* < .05. ** *p* < .01. *** *p* < .001.
(DOCX)

S3 Table Mixed ANOVAs for group and task effects across measures. ANOVA = Analyses of variance. *** *p* < .001.
(DOCX)

S4 Table Descriptive statistics and group comparisons for children and adults across tasks and measures, controlling for task duration. *** *p* < .001.
(DOCX)
