# Supplementary material for: Are children really more creative than adults? A mixed-methods examination of creative performance and experience
Source: PLoS One. 2026 Oct 21;21(10):e0346553. doi: 10.1371/journal.pone.0346553 (PMC13686510; doi:10.1371/journal.pone.0346553)
Supplement: Supplementary file 1 [file pone.0346553.s001.docx]

**S1 Table. Interrater reliability [ICC(3, 5)] of scores across creativity tasks.** Analyses of variance-based 95% CIs are reported in brackets.

|  | Novelty | Effectiveness | Creativity |
| --- | --- | --- | --- |
| Alternate Uses Task | .845 [.825, .862] | .789 [.762, .813] | .864 [.847, .880] |
| Structured Creative Drawing | .977 [.935, .997] | .980 [.944, .998] | .961 [.892, .995] |
| Free Drawing | .975 [.929, .997] | .945 [.846, .993] | .972 [.922, .997] |
