# Supplementary material for: Are children really more creative than adults? A mixed-methods examination of creative performance and experience
Source: PLoS One. 2026 Oct 21;21(10):e0346553. doi: 10.1371/journal.pone.0346553 (PMC13686510; doi:10.1371/journal.pone.0346553)
Supplement: Supplementary file 2 [file pone.0346553.s002.docx]

**S2 Table. Response-level correlations among novelty, effectiveness, and overall creativity ratings across creativity tasks.** * *p* < .05. ** *p* < .01. *** *p* < .001.

| Task | *r* Novelty, Effectiveness | *r* Novelty, Creativity | *r* Effectiveness, Creativity |
| --- | --- | --- | --- |
| Alternate Uses Task | -.09* | .92*** | .12** |
| Structured Creative Drawing | .90*** | .96*** | .96*** |
| Free Drawing | .39*** | .78*** | .83*** |
