# Supplementary material for: Are children really more creative than adults? A mixed-methods examination of creative performance and experience
Source: PLoS One. 2026 Oct 21;21(10):e0346553. doi: 10.1371/journal.pone.0346553 (PMC13686510; doi:10.1371/journal.pone.0346553)
Supplement: Supplementary file 3 [file pone.0346553.s003.docx]

**S3 Table. Mixed ANOVAs for group and task effects across measures.** ANOVA = Analyses of variance. *** *p* < .001.

| Measure | | *F* | *df* | *η^2^* |
| --- | --- | --- | --- | --- |
| Novelty | |  |  |  |
|  | Group | 120.21*** | 1, 160 | 0.319 |
|  | Condition | 237.95*** | 1, 160 | 0.359 |
|  | Group x Condition | 176.68*** | 1, 160 | 0.293 |
| Effectiveness | |  |  |  |
|  | Group | 352.46*** | 1, 160 | 0.606 |
|  | Condition | 3.87 | 1, 160 | 0.007 |
|  | Group x Condition | 38.18*** | 1, 160 | 0.067 |
| Creativity | |  |  |  |
|  | Group | 243.13*** | 1, 160 | 0.499 |
|  | Condition | 105.00*** | 1, 160 | 0.184 |
|  | Group x Condition | 126.83*** | 1, 160 | 0.214 |
| Duration [min] | |  |  |  |
|  | Group | 15.30*** | 1, 160 | 0.051 |
|  | Condition | 0.75 | 1, 160 | 0.002 |
|  | Group x Condition | 49.96*** | 1, 160 | 0.121 |
| Motivation | |  |  |  |
|  | Group | 28.80*** | 1, 159 | 0.116 |
|  | Condition | 6.40 | 1, 159 | 0.011 |
|  | Group x Condition | 18.86*** | 1, 159 | 0.031 |
| Enjoyment | |  |  |  |
|  | Group | 20.29*** | 1, 159 | 0.065 |
|  | Condition | 1.49 | 1, 159 | 0.004 |
|  | Group x Condition | 15.82*** | 1, 159 | 0.043 |
| Self-Efficacy | |  |  |  |
|  | Group | 116.60*** | 1, 159 | 0.344 |
|  | Condition | 2.39 | 1, 159 | 0.004 |
|  | Group x Condition | 2.00 | 1, 159 | 0.004 |
| Self-Appraisal | |  |  |  |
|  | Group | 211.91*** | 1, 158 | 0.429 |
|  | Condition | 1.85 | 1, 158 | 0.005 |
|  | Group x Condition | 9.39 | 1, 158 | 0.025 |
| Anxiety | |  |  |  |
|  | Group | 10.01 | 1, 159 | 0.048 |
|  | Condition | 0.04 | 1, 159 | 0.000 |
|  | Group x Condition | 0.37 | 1, 159 | 0.000 |
