# Supplementary material for: Are children really more creative than adults? A mixed-methods examination of creative performance and experience
Source: PLoS One. 2026 Oct 21;21(10):e0346553. doi: 10.1371/journal.pone.0346553 (PMC13686510; doi:10.1371/journal.pone.0346553)
Supplement: Supplementary file 4 [file pone.0346553.s004.docx]

**S4 Table. Descriptive statistics and group comparisons for children and adults across tasks and measures, controlling for task duration.**
*** *p* < .001.

| Measure | Task |  | *Children* | | *Adults* | | *t* | *df* | Cohen’s *d* |
| --- | --- | --- | --- | --- | --- | --- | --- | --- | --- |
|  |  |  | *M* | *SD* | *M* | *SD* |  |  |  |
| Novelty | Alternate Uses Task | 1-5 | 2.79 | 0.61 | 3.67 | 0.36 | 8.23*** | 157.00 | -1.128 |
|  | Free Drawing | 1-5 | 2.30 | 0.73 | 2.36 | 0.71 | 1.27 | 159.00 | -0.201 |
|  | Structured Drawing | 1-5 | 2.55 | 0.65 | 4.29 | 0.49 | 11.18*** | 159.00 | -1.140 |
| Effectiveness | Alternate Uses Task | 1-5 | 4.23 | 0.56 | 4.86 | 0.18 | 6.91*** | 157.00 | -0.958 |
|  | Free Drawing | 1-5 | 1.87 | 0.55 | 3.05 | 0.83 | 13.74*** | 159.00 | -2.157 |
|  | Structured Drawing | 1-5 | 1.69 | 0.47 | 3.52 | 0.56 | 14.66*** | 159.00 | -1.372 |
| Creativity | Alternate Uses Task | 1-5 | 2.87 | 0.60 | 3.86 | 0.32 | 9.93*** | 157.00 | -1.338 |
|  | Free Drawing | 1-5 | 2.02 | 0.61 | 2.61 | 0.74 | 7.72*** | 159.00 | -1.218 |
|  | Structured Drawing | 1-5 | 2.05 | 0.59 | 3.92 | 0.51 | 13.56*** | 159.00 | -1.305 |
| Self-Efficacy | Alternate Uses Task | 1-5 | 3.54 | 1.50 | 2.97 | 1.13 | -2.70 | 158.00 | 0.383 |
|  | Free Drawing | 1-5 | 4.51 | 1.05 | 2.81 | 1.15 | -9.57*** | 158.00 | 1.514 |
|  | Structured Drawing | 1-5 | 4.53 | 1.00 | 3.10 | 1.13 | -6.91*** | 158.00 | 0.759 |
| Motivation | Alternate Uses Task | 1-5 | 4.02 | 1.26 | 3.40 | 1.08 | -4.27*** | 158.00 | 0.600 |
|  | Free Drawing | 1-5 | 4.52 | 0.90 | 3.39 | 1.13 | -6.92*** | 158.00 | 1.096 |
|  | Structured Drawing | 1-5 | 4.41 | 1.13 | 4.03 | 1.01 | -2.94 | 158.00 | 0.344 |
| Enjoyment | Alternate Uses Task | 1-5 | 3.96 | 1.21 | 3.21 | 0.93 | -3.93*** | 158.00 | 0.554 |
|  | Free Drawing | 1-5 | 4.62 | 0.77 | 3.70 | 1.11 | -5.98*** | 158.00 | 0.948 |
|  | Structured Drawing | 1-5 | 4.38 | 1.08 | 4.29 | 0.84 | -2.21 | 158.00 | 0.260 |
| Self-Appraisal | Alternate Uses Task | 1-5 | 3.97 | 1.26 | 3.13 | 0.91 | -4.59*** | 157.00 | 0.644 |
|  | Free Drawing | 1-5 | 4.76 | 0.70 | 2.77 | 1.26 | -12.57*** | 157.00 | 1.992 |
|  | Structured Drawing | 1-5 | 4.63 | 0.78 | 3.26 | 1.12 | -7.94*** | 157.00 | 0.859 |
| Anxiety | Alternate Uses Task | 1-5 | 1.52 | 1.04 | 2.20 | 1.12 | 3.67*** | 158.00 | -0.518 |
|  | Free Drawing | 1-5 | 1.34 | 0.93 | 1.84 | 1.20 | 2.83 | 157.00 | -0.450 |
|  | Structured Drawing | 1-5 | 1.40 | 0.99 | 1.81 | 1.05 | 1.94 | 158.00 | -0.228 |
