# Supplementary material for: Are children really more creative than adults? A mixed-methods examination of creative performance and experience
Source: PLoS One. 2026 Oct 21;21(10):e0346553. doi: 10.1371/journal.pone.0346553 (PMC13686510; doi:10.1371/journal.pone.0346553)
Supplement: Supplementary file 5 [file pone.0346553.s006.docx]

**Point-by-Point Reviewer Response**

Dear Editor, dear Reviewers,

Thank you very much for reviewing our manuscript entitled *Are children really more creative than adults? A mixed-methods examination of creative performance and experience* and for your thorough examination of the manuscript and for providing constructive and insightful feedback on how to improve it. Please find below a detailed description of how each Reviewer point was addressed in the revised manuscript. We believe that careful consideration of the thoughtful feedback has helped to strengthen the article substantially.

In addition, we carefully revised the manuscript to ensure full compliance with the journal’s formatting and reporting requirements.

Sincerely,

The Authors

**Reviewer #1**

**I would like to thank for the opportunity to review this manuscript. Please see the following comments to consider to further increase the quality of this manuscript.**

**The manuscript entitled “Are children really more creative than adults? A mixed-methods examination of creative performance and experience” addresses a timely and conceptually important question by integrating qualitative and quantitative approaches. The study is clearly structured, theoretically grounded, and methodologically transparent, and it makes a valuable contribution by disentangling creative performance from experiential and motivational aspects of creativity. The combination of a qualitative pre-study with an experimental comparison between preschool children and adults is particularly commendable, as it allows the authors to bridge lay beliefs and empirical evidence in a coherent manner.**

**At the same time, several aspects of the manuscript could be strengthened to enhance its theoretical precision, methodological rigor, and overall clarity.**

**The introduction is generally well-written and situates the study within relevant literature; however, the theoretical framing could be sharpened further. Please consider clarifying more explicitly what is meant by “creativity” at different points in the manuscript, as the argument currently moves between divergent thinking, creative potential, creative behavior, and subjective experience without always clearly distinguishing these constructs. Although the authors acknowledge the multidimensionality of creativity, it would be beneficial to articulate a more explicit conceptual model early in the introduction that specifies how cognitive performance, affective-motivational experience, and self-beliefs are theoretically related. This would help readers better understand how the different measures used in the study map onto the broader construct of creativity.**

**Perhaps the authors could also engage more critically with alternative frameworks, such as the distinction between “little-c” and “Big-C” creativity or the 4C model, to further situate their contribution.**

Response: We agree that more background on the diverse facets of creativity and their interplay is helpful to better contextualize the measures of this study. We revised and extended the relevant section in the literature accordingly:

“Creativity is a multifaceted construct that extends beyond creative thinking ability to include relevant traits, skills, and behavioral components. Specifically, creative behavior and achievement is known to develop and arise from the interplay of cognitive abilities (e.g., DT ability), core personality traits (e.g., openness to new experiences), creative self-beliefs (e.g., creative confidence) and expertise in the context of environmental influences [15,16]. Some of these facets, such as domain-specific expertise and lifetime creative achievement, depend on accumulated knowledge and prolonged practice and therefore typically increase with age, favoring adults [17]. For example, professional or eminent levels of creativity (i.e., Pro-c and Big-C) are typically observed only at later career stages or even assigned posthumous [18,19]. Other facets may align more closely with popular intuitions about children’s creativity. Children may engage with creative tasks in a more play-like manner [20,21] reflecting high openness [22], and intrinsic motivation [23], thus enjoying the task without being overly concerned about the outcome [24]. Adults, in contrast, are more aware of their strengths and weaknesses [25] and typically more sensitive to external expectations and social evaluation [26], which may support goal-directed performance via metacognition and self-regulation [27,28] but also increase worries and constrain risk-taking [29,30]. Considering both cognitive and non-cognitive aspects of creativity therefore appears essential when examining whether children are more or less creative than adults.”

**The rationale for the qualitative pre-study is convincing, yet its integration into the overall research design could be elaborated. While the manuscript states that the qualitative findings informed the design of the main study, the exact pathways of this influence remain somewhat implicit. Please consider providing more concrete examples of how specific themes identified in the interviews translated into design choices, such as the selection of tasks or the inclusion of particular self-report measures.**

Response: While the manuscript already indicated that the qualitative pre-study informed the design of the main study, we agree that this link can be made more explicit. We therefore revised and expanded the section on design implications accordingly:

### **“Design implications for the main study**

Qualitative findings suggested that children’s creativity is commonly associated with openness, spontaneity, intrinsic motivation, and freedom from external constraints, whereas adults’ creativity was primarily associated with goal orientation, accumulated knowledge and experience, and domain specificity. Concrete expressive behaviors (e.g., drawing, storytelling, experimenting) were mentioned for both groups but contextualized differently: children’s creativity was typically described in terms of playful exploration, spontaneous experimentation, and low inhibitions, whereas adults’ creativity was more often characterized as more product- and goal-oriented, and situated within specific domains such as profession or hobbies. These insights motivated us to examine creative performance under two conditions that might favor children versus adults [20, but see 34], respectively: 1) an unconstrained, play-like condition that does pose a clear goal or time-limit (cf. free drawing task), and 2) a constrained, test-like condition that has a clear goal and time-limit (cf. structured drawing task). Additionally, we included the well-established alternative uses task for reference with the rich literature using this task [35] and scored creative performance independently for novelty, effectiveness and overall creativity [36], as children’s playful exploration behavior might yield more novel yet less effective responses.

Environmental references centered on freedom, with children frequently described as having fewer external constraints and greater latitude for exploration, while adults were portrayed as facing time pressure, social expectations, and performance demands. Therefore, we decided to measure subjective experience during the creative tasks besides actual task performance. Finally, since participants predominantly located peak creativity in early childhood, particularly shortly before school entry, we decided to focus on preschool children in the comparison with adults.”

**Additionally, the qualitative sample is relatively small (N = 10), which is not uncommon but nevertheless limits the breadth of perspectives captured. It would be helpful to acknowledge this more explicitly and perhaps justify why this sample size was considered sufficient for the purposes of the study.**

Response: We agree that the qualitative sample size may seem relatively small and appreciate the opportunity to clarify this point. While a sample size of N = 10 limits the breadth of perspectives captured, it is consistent with methodological recommendations for achieving thematic saturation in interview-based research. Recent reviews suggest that saturation is often reached within approximately 9-17 interviews (Hennink & Kaiser, 2022) or even around 9 interviews for theme-based analyses (Wutich et al., 2024).

Importantly, the aim of the qualitative pre-study was not to provide an exhaustive account of lay beliefs, but rather to identify central and recurring themes that could inform the design of the subsequent quantitative study. In this context, the sample size was considered sufficient to capture the most salient intuitions relevant to our research aims.

We have now made this rationale more explicit in the manuscript:

“10 adult laypeople (60% female) with a mean age of 29.70 years (*SD* = 2.36) participated in semi-structured interviews designed to elicit beliefs about creativity in children and adults after giving verbal informed consent. Participants were recruited via convenience sampling from the local community and the researchers’ personal networks. Interviews addressed perceived developmental changes in creativity (including the age at which children are considered the most creative) and beliefs about characteristics and behaviors associated with creativity in children versus adults. Interviews were audio-recorded, transcribed verbatim, and analyzed using qualitative content analysis. The sample size was consistent with recommendations for achieving thematic saturation in interview-based research [31,32] and appropriate for the exploratory aim of the pre-study.”

Hennink, M., & Kaiser, B. N. (2022). Sample sizes for saturation in qualitative research: A systematic review of empirical tests. *Social science & medicine*, *292*, 114523. <https://doi.org/10.1016/j.socscimed.2021.114523>

Wutich, A., Beresford, M., & Bernard, H. R. (2024). Sample sizes for 10 types of qualitative data analysis: an integrative review, empirical guidance, and next steps. *International Journal of Qualitative Methods*, *23*, 16094069241296206. <https://doi.org/10.1177/16094069241296206>

**The methodology of the quantitative study is generally robust and well-described, with clear procedures and open science practices (e.g., OSF repository) that are highly commendable. However, several methodological considerations merit further attention.**

**First, the use of a fixed task order may introduce order or fatigue effects, particularly given the age differences between groups. Please consider discussing this limitation more explicitly and, if possible, justifying why counterbalancing was not implemented.**

Response: The free drawing task preceded the structured drawing task because it was framed as a familiarization with the task materials. The AUT could have been counterbalanced relative to the drawing tasks, but we always kept it first as we did not plan to compare performance between these tasks, but we agree that this approach does not control for carry-over and time effects. We now discuss this as a limitation:

“Fourth, other creativity tasks (e.g., insight problem-solving) could be included and task order should be counterbalanced to limit fatigue effects.”

**Second, while the adaptation of tasks for children is appropriate, the comparability of measures across age groups remains a potential concern. For instance, the reliance on self-report measures in young children may raise questions about validity, as also briefly acknowledged in the discussion. It might strengthen the manuscript to elaborate on how these measures were validated or adapted for this age group and to what extent measurement invariance can be assumed.**

Response: We now provide more information on how measures were adapted to the young target group, informed by cognitive surveys with two young girls.

“The feasibility of the procedure was initially tested in a cognitive survey involving two children (four and seven years old), which informed final task adjustments. “

“To account for the children’s limited experience with the object, an actual tin can was provided to all participants (children and adults), allowing them to explore it during the task.”

“Following similar assessments with young children, we used brief items that were communicated orally in an age-appropriate language using a five-point scale anchored with emoticons [e.g., 38]. For each item, all response options were described by the experimenter, and the question was rephrased if necessary.”

The performance measures were found to work well as responses were task-relevant (e.g., the structured drawing task resulted in recognizable fantasy animals). The single item measures prevented us from running proper analyses of measurement invariance. For self-report questions, children sometimes required a rephrasing of the question. Despite all measures of precaution, we cannot rule out that preschool children have a different understanding of the constructs and differ in their general use of the response scale. Therefore, we discuss these important points as study limitations.

**The scoring of creative performance is a strength of the study, particularly the use of multiple raters and separate ratings of novelty and effectiveness. Nevertheless, please consider providing more detail on the training of raters and the rating procedure, as this would enhance transparency and reproducibility.**

Response: We have now clarified the rater training and rating procedure in the Methods section. Specifically, we added that the initial training involved familiarization with the rating scales and criteria, followed by supervised practice on a subset of responses and drawings, including discussion of uncertainties, before the remaining responses were evaluated independently.

**In addition, the choice of the “max-3” scoring approach for the AUT is justified by prior work, but it might be useful to briefly explain its implications for interpretation, especially for readers less familiar with divergent thinking methodology.**

Response: We agree that a clarification of the rationale for using the max-3 scoring approach is warranted. We have therefore expanded the description in the Methods section to clarify that this approached reduced confounding effects of ideational fluency by focusing on the quality of participants’ best three ideas. Specifically, max-3 scores are based on the average ratings of the three highest-rated responses per participant:

“For the AUT, a max-3 aggregation approach was applied to reduce response fluency confounds by focusing on a fixed number of responses; specifically, scores reflect the average ratings of the three highest-rated responses per participant [39].”

**The statistical analysis is appropriate overall, but some aspects could be clarified. The manuscript reports a stringent significance threshold (p < .001), which is commendable in terms of controlling Type I error, yet it would be helpful to provide a clearer rationale for this decision and to discuss its implications for statistical power.**

Response: We agree that the choice of a stringent significance threshold requires clarification. Given the number of statistical tests conducted, the use of a conservative threshold (*p* < .001) appears justified, as it approximates the level of stringency that would result from formal multiple comparison corrections (e.g., Bonferroni-type adjustments). Rather than applying varying corrections across analyses, we opted for a uniform threshold to ensure transparency and consistency. Such stringent thresholds have also been discussed in the literature to strengthen the evidential value of findings (e.g., Benjamin et al., 2018). We have clarified this rationale in the manuscript:

“All tests were two-tailed. To account for multiple comparisons, a conservative significance threshold of *p* < .001 was applied. This threshold approximated the level of stringency achieved by common correction procedures and ensures a consistent criterion across analyses.”

Benjamin, D. J., Berger, J. O., Johannesson, M., Nosek, B. A., Wagenmakers, E. J., Berk, R., ... & Johnson, V. E. (2018). Redefine statistical significance. *Nature Human Behaviour*, *2*(1), 6-10. <https://doi.org/10.1038/s41562-017-0189-z>

**Furthermore, while effect sizes are reported, their interpretation could be expanded in the results section to aid readers in understanding the practical significance of the findings. Perhaps the authors could also consider complementing the frequentist analyses with confidence intervals more consistently in the main text, not only in the supplementary materials.**

Response: To improve the interpretation of our findings, we now report 95% confidence intervals alongside all key statistics in the main text, thereby facilitating a clearer understanding of their practical significance.

**The results are clearly presented and logically organized, and the figures (e.g., Figures 2 and 3) effectively illustrate group differences across tasks and measures (see pages 27–28). However, the narrative could be made slightly more concise by reducing some repetition between text and figures. At the same time, it might be beneficial to highlight key interaction effects more explicitly, particularly regarding how task structure moderates differences between children and adults.**

Response: We now discuss the significant interactions more explicitly:

“Significant interactions for all three performance measures indicated that smaller group differences were observed for novelty and creativity in the unstructured drawing, whereas group differences in effectiveness were highest in the structured drawing task.”

“Significant interactions for motivation and enjoyment indicated that adults generally felt more comfortable in the structured drawing task, whereas children enjoyed the free drawing task more.”

**The discussion provides a thoughtful interpretation of the findings and successfully reconciles the apparent discrepancy between lay beliefs and empirical evidence. The distinction between creative performance and creative engagement is a particularly valuable contribution. Nevertheless, some interpretations could be tempered to avoid overgeneralization. For example, the conclusion that children’s advantage lies in “creative agency” is plausible but somewhat speculative, given that agency was not directly measured. Please consider framing such claims more cautiously and grounding them more explicitly in the data.**

Response: We agree that the early reference to creative agency in the discussion came a bit out of the blue. We removed this early reference and introduce this notion more carefully with reference to the *Creative Behavior as Agentic Action* model by Karwowski and Beghetto (2019):

“Following the *Creative Behavior as Agentic Action* model [47], creative behavior is not just a function of ability, but also of creative confidence beliefs. In our study, children exhibited lower creative thinking potential than adults, yet higher creative confidence and overall higher creative agency, in terms of their intrinsic willingness to engage in creative behavior.”

**The limitations section is appropriate and acknowledges several important issues, yet it could be expanded further. In particular, the cross-sectional design limits developmental inferences, and this could be discussed in more depth. Additionally, cultural factors may influence both lay beliefs and creative expression, and it might be worthwhile to note that the sample is drawn from a specific cultural context, which may limit generalizability.**

Response: We fully agree that both the cross-sectional design and the cultural context of the sample limit the generalizability of the findings. We therefore revised the limitations section to acknowledge these points more explicitly:

“First, cross-sectional comparisons are susceptible to cohort effects and do not enable causal inference.”

“Finally, the findings are based on samples drawn from a specific cultural context. Future research could examine how lay beliefs and creative expression vary across different populations and backgrounds.”

**The manuscript could be further strengthened by more explicitly integrating a self-determination theory (SDT) perspective to interpret the observed differences in creative engagement and experience. In particular, Ahmadi et al. (2023) provide a comprehensive and systematically developed classification of motivational behaviors that support or thwart the basic psychological needs of autonomy, competence, and relatedness, thereby shaping individuals’ motivation and engagement. Please consider using this framework to more precisely conceptualize children’s higher motivation, enjoyment, and confidence as indicators of need-supportive contexts, and adults’ comparatively higher anxiety as potentially linked to need-thwarting conditions. Such integration could offer a more theoretically grounded explanation of why children appear more “creative” in terms of engagement, and perhaps clarify how contextual factors and task structures influence creative expression across age groups.**

**Ahmadi, A., Noetel, M., Parker, P., Ryan, R. M., Ntoumanis, N., Reeve, J., Beauchamp, M., Dicke, T., Yeung, A., Ahmadi, M., Bartholomew, K., Chiu, T. K. F., Curran, T., Erturan, G., Flunger, B., Frederick, C., Froiland, J. M., González-Cutre, D., Haerens, L., … Lonsdale, C. (2023). A classification system for teachers’ motivational behaviors recommended in self-determination theory interventions. Journal of Educational Psychology, 115(8), 1158–1176.** [**https://doi.org/10.1037/edu0000783**](https://doi.org/10.1037/edu0000783)

Response: We seized this suggestion and now added an interpretation of our findings in the context of the motivational behaviors in the context of SDT:

“This heightened agency in children can also be interpreted through the lens of *Self-Determination Theory* and relevant motivational behaviors [48]. Children’s higher enjoyment and confidence could be seen as indicators of need-supportive contexts, where the basic psychological needs for autonomy and competence are satisfied, likely because children perceive creative tasks as low-stakes, playful activities [49]. Conversely, the adults’ higher anxiety suggests need-thwarting conditions—where evaluative pressure or rigid self-expectations may undermine their autonomy and competence.”

**Reviewer #2**

**This study by Saretzki et al. investigates different aspects of creativity in preschool children and adults. It is an interesting study, but many clarifications are needed. The main weakness is the lack of information on participants and participant selection, which limits interpretations, in my opinion. I detailed my concerns below. But I really appreciate that all data and questionnaires are open-source.**

**Please provide references for the statement, lines 34-37: “At the same time, this believe appears to contrast…”.**

Response: We now support this statement with relevant references:

At the same time, this belief appears to contrast with evidence from age-normed creativity tests and meta-analytical work, which typically show a steady increase in creative thinking performance from childhood into adulthood [4–7].

**For the qualitative study, I am unsure how much this brings valid results, as the sample of 10 individuals is really low. Further, no information is provided on how these 10 people got selected. Given the small sample, any interpretations can be easily biased based on potential disorders, age, culture, education level and, maybe more importantly, if they have children (I believe that could make a big difference in overall beliefs about children’s creativity). I think, my main question is why should I trust their opinion to know anything about children’s creativity? Any discussion/results related to this qualitative study should be more cautious in their validity and suggest that these results should not be generalized.**

Response: We agree that the qualitative pre-study is based on a sample that may seem relatively small and non-representative and is therefore not intended to support generalizable conclusions. Consistent with our response to a similar comment by Reviewer 1, we now justify the sample size in more detailby noting that it aligns with methodological recommendations for interview-based research and with evidence suggesting that thematic saturation is often reached within approximately 9-17 interviews or even around 9 interviews for theme-based analyses (Hennink & Kaiser, 2022; Wutich et al., 2024).

Importantly, the aim of the pre-study was not to provide valid estimates of children’s creativity, but to integrate the perspective of lay people to inform the design of the quantitative main study. Participants were recruited via convenience sampling for this exploratory purpose. We have clarified the recruitment procedure in the Methods section and added a corresponding limitation emphasizing the restricted generalizability of the findings and their potential dependence on participants’ backgrounds.

**While everything is provided on OSF, more details on the questionnaires should still be provided in the method as the paper should stand on its own.**

Response: We agree that the manuscript should provide sufficient detail to understand the assessed constructs and procedures independently of supplemental materials. In the revised version, we have therefore added more details on creative thinking tasks and the self-report measures, including necessary adaptations for the young age group. For example, we describe that self-report measures used brief, age-appropriate items, oral presentations, and a five-point response scale anchored with emoticons that were individually described by the experimenter. We also provide the questions of single self-report items in the main text.

**For the quantitative study, inclusion/exclusion criteria should be provided for the participants. What about neurodevelopmental disorders? Where the participants all considered healthy/typically developing? If disorders and developmental delays were not considered (especially in the children, but it also matters in the adult group), it is a major limitation as it is likely that ADHD or autism may impact the level of creativity or attention needed to complete the tasks. How did these participants get recruited? Without this information, I don’t believe that results can be fully interpreted.**

Response: We agree that it is helpful to offer more information on the sample recruiting strategy. This study aimed for largely representative samples within the target age groups. A comprehensive developmental and clinical assessment for all participants was not feasible in the context of this study. Children with severe, clinical conditions would, however, typically not attend the public kindergarten. It should also be noted that the relationship between creativity and subclinical conditions were found to be subtle and complex, and not necessarily negative (e.g., Boot et al., 2017; Hoogman et al., 2020). In sum, we believe that it is unlikely that the consistent performance differences observed between children and adults are due to neurodevelopmental disorders. We now provide more information on the recruitment of participants:

“Children were recruited from nine local kindergartens in their final year prior to school entry. Adults were recruited from the general population. The study aimed for largely representative samples within preschoolers and adults and therefore did not apply other formal inclusion/exclusion criteria.”

Boot, N., Nevicka, B., & Baas, M. (2017). Subclinical symptoms of attention-deficit/hyperactivity disorder (ADHD) are associated with specific creative processes. *Personality and Individual Differences*, *114*, 73–81. <https://doi.org/10.1016/j.paid.2017.03.050>

Hoogman, M., Stolte, M., Baas, M., & Kroesbergen, E. (2020). Creativity and ADHD: A review of behavioral studies, the effect of psychostimulants and neural underpinnings. *Neuroscience & Biobehavioral Reviews*, *119*, 66-85. <https://doi.org/10.1016/j.neubiorev.2020.09.029>

**Related to my previous comment, my other major concern is how did the investigators ensure that the children understood all the questions and the concept of being anxious/worried, or the concept of “creativity”, especially in the youngest children (Some were only 4!), or in children that may have developmental delay (if this was not recorded). It is therefore possible that the group differences noted were not due to development per se, but due to disorders, lack of understanding, lack of being aware of their own anxiety (because they are too young to understand it), etc.**

Response: We agree that fair group comparisons rely on proper understanding of the tasks and scales, especially for the young age group. We now provide additional details on the measures taken to this end, including cognitive surveys, the use of age-appropriate language, oral instructions and responses and, if needed, additional explanations:

“To account for the children’s limited experience with the object, an actual tin can was provided to all participants (children and adults), allowing them to explore it during the task. Responses were given orally, recorded, and later transcribed, consistent with established procedures for assessing creativity in young children [5].”

“Following similar assessments with young children, we used brief items that were communicated orally in an age-appropriate language using a five-point scale anchored with emoticons [e.g., 38]. For each item, all response options were described by the experimenter, and the question was rephrased if necessary.”

The performance measures showed face validity as responses were task-relevant (e.g., the structured drawing task resulted in recognizable fantasy animals). For self-report questions, children sometimes required a rephrasing of the question. Despite all employed measures, we cannot rule out that preschool children have a different understanding of concepts like creativity or emotions and also differ in their general use of the response scale. Therefore, we discuss these points as important study limitations and advise that findings need to be interpreted cautiously in their context.

**The age range of the adults recruited was rather large (30 years). As for developmental effects of age on creativity, it is known that aging is associated with changes in creativity. It does not seem that there was any consideration for potential age effect within the adult groups. It could cause increased variability and reduce effect sizes.**

Response: Most of the adult participants were young adults (mean age = 25.31, *SD* = 5.90). To our knowledge, creativity is fairly stable in the young adult age range, and most pronounced developmental effects are observed either during childhood or late age. We now provide a more specific age description by clarifying for adults that “85% were between 20 and 30 years”.

**Please provide a reference for the AUT.**

Response: We note that the AUT is not a single standardized instrument with a definitive reference, but rather a widely used divergent thinking paradigm that varies in its administration and scoring across studies (see also Saretzki et al., 2026). To improve clarity and provide a conceptual anchor, we have now added a reference to Guilford (1967), who introduced such tasks in his seminal work on divergent thinking.

**Given the very restricted age range of the children included in the study, I think the discussion should be more precise and not generalized to “children and adults” as it is likely going to change if they had 10 years old. While this is acknowledged in the limitation, it should be more precise through the discussion (e.g., “preschool children” vs “children”). Otherwise, I think the interpretations are over-stating the findings.**

Response: We agree that the age range of the children sample should be reflected accurately. We have therefore revised relevant sections to refer specifically to preschool children rather than children in general. For example, methods and results clearly introduce the sample as preschool children but also use the shorter term “children” occasionally to avoid repetitive language. We thoroughly revised the discussion to specify that findings refer to preschool children and only retained the broader term “children” in places where it refers to common beliefs or prior literature. These revisions ensure that the scope of our conclusions is appropriately bounded and does not overgeneralize beyond the studied age group.
