# Supplementary material for: Are children really more creative than adults? A mixed-methods examination of creative performance and experience
Source: PLoS One. 2026 Oct 21;21(10):e0346553. doi: 10.1371/journal.pone.0346553 (PMC13686510; doi:10.1371/journal.pone.0346553)
Supplement: Supplementary file 6 [file pone.0346553.s007.docx]

**Point-by-Point Reviewer Response**

Dear Editor, dear Reviewers,

Thank you very much for the opportunity to submit a revised version of our manuscript entitled *Are children really more creative than adults? A mixed-methods examination of creative performance and experience*. We are happy that the original two reviewers were already satisfied with the previous version and are grateful for the additional constructive and insightful comments provided during this review round.

Please find below a detailed description of how each Reviewer point was addressed in the revised manuscript. We believe that careful consideration of the thoughtful feedback has helped to strengthen the article substantially.

For ease of review, text changes implemented during the first revision are highlighted in yellow, while changes introduced in the present revision are highlighted in pink throughout the marked manuscript.

Sincerely,

The Authors

**Reviewer #3**

**I found this to be a thoughtful and conceptually interesting paper that makes a worthwhile contribution to the creativity literature. In particular, I valued the authors’ attempt to disentangle creative performance from creative engagement, and to empirically examine why children are often perceived as “more creative” despite adults outperforming them on externally evaluated creative tasks. The overall pattern of findings was coherent and theoretically generative, especially the distinction between children’s stronger affective engagement (e.g., higher confidence, enjoyment, and intrinsic motivation) and adults’ stronger performance under evaluative or goal-directed conditions. I also appreciated the inclusion of lower-constraint tasks within the design, which strengthened the study by moving beyond purely structured creativity assessments. Overall, I found the paper clearly written, theoretically engaged, and addressing a genuinely interesting and culturally resonant question.**
Response: We thank the reviewer for these positive and encouraging comments and are pleased that the contribution and rationale of our study were appreciated.

**Additional reflections and future directions
One avenue that may be fruitful for future research concerns forms of creativity that are less product-oriented and less tightly tied to externally evaluated performance. While the inclusion of lower-constraint tasks strengthens the present design, many everyday perceptions of children’s creativity may emerge from more socially situated and play-based contexts, such as pretend play, collaborative imaginative activity, narrative improvisation, or spontaneous symbolic exploration. Investigating creativity in such settings may help further clarify the distinction observed here between creative performance and creative engagement/agency. For example, in genuinely open-ended collaborative imaginative contexts, adults may demonstrate advantages in sustaining coherent narratives, integrating abstraction or humour, and collectively scaffolding complex symbolic worlds, even while children appear more spontaneous, playful, or intrinsically immersed in the activity itself.**
Response: Thank you for this valuable suggestion. We agree that future research should compare children and adults across a broader range of creative activities and consider additional indicators of creative expression beyond conventional performance measures. We have incorporated this perspective into the *Limitations and future directions* section:

“Fourth, other creativity tasks (e.g., insight problem-solving) and more open-ended or collaborative forms of creative activity could be included, and task order should be counterbalanced to limit fatigue effects. Future research may also consider a broader range of evaluation criteria, such as narrative coherence and collaborative idea development when comparing creativity across age groups.”

**The findings also seem to create a natural opportunity for a qualitative follow-up study with the same adult participants. A particularly interesting feature of the present design is that the adult participants both completed the creativity tasks and, as a group, strongly endorsed the view that children are more creative. This places them in a unique position to help unpack the apparent disconnect between everyday beliefs about children’s creativity and the pattern of task-based performance reported in the study. For example, participants could be presented with the study’s findings and invited to reflect on whether the results surprised them, whether they viewed the administered tasks as representative of creativity more broadly, and whether their perceptions of children’s creativity are grounded more in observable engagement, spontaneity, playfulness, or imaginative expression than in externally evaluated creative products. Such a follow-up could provide particularly rich insight into some of the interpretive tensions the paper itself raises regarding the relationship between perceived creativity, creative engagement, and creative performance.**

Response: Thank you for this great suggestion. We have incorporated this idea into the Future Directions section:

“Finally, as the findings are based on samples drawn from a specific cultural context, future research could examine how lay beliefs and creative expression vary across different populations and background. Yet, another avenue for future work would be to confront adults viewing children as more creative with the findings of this study and ask them to justify or elaborate their views in the light of these results.”

**Reviewer #4
The study is well-structured and motivated, and it provides a valuable contribution to the creativity literature. Nevertheless, several aspects should be addressed to strengthen the manuscript and improve its overall impact.**

**1. As a general comment, the resolution of figures should be improved.**

Response: Thank you for this comment. We re-examined all figures and confirmed that they were submitted in accordance with the journal’s requirements. We acknowledge that the figure quality is sometimes degraded in the version that is shared with the reviewers, while often providing the option to retrieve a high-resolution version via a link.

**2. Based on the methods and terminology used throughout the manuscript, the title should explicitly indicate that the study was conducted on preschool children.**

Response: Thank you for this suggestion. We agree that the specific age groups should be clearly communicated and have therefore clarified throughout the manuscript that the child sample consisted of preschool children. However, we chose to retain the original title because this work addressed the well-established myth of whether children are more creative than adults. The study eventually operationalized children by preschool children, because the qualitative study suggested that differences in creativity might be most pronounced in the preschool age group. For this reason, we would prefer to maintain the original title, especially after specifying the children group in all other relevant places.

**3. I believe that these qualitative preliminary studies are important for science progress. Was this qualitative study developed within a citizen science framework? Did the experimental design or questions evolve as a consequence of the interviews?**

Response: Thank you for this interesting question. The qualitative interviews were not conducted within a formal citizen science framework, as participants contributed solely as research participants and were not involved in the design or conduct of the research. However, the qualitative phase was central to the project and directly informed the design of the quantitative study. As described in the subsection *Design implications for the main study*, the interview findings guided several key design decisions, including the selection of task conditions, outcome measures, and the focus on preschool children.

**4. Were the semi-structured interviews conducted individually or in groups?**

Response: The interviews were conducted individually. We have clarified this in the manuscript:

“10 adult laypeople (60% female) with a mean age of 29.70 years (*SD* = 2.36) participated in individual semi-structured interviews designed to elicit beliefs about creativity in children and adults after giving verbal informed consent.”

**5. More information about the laypeople would help for readers understand their contribution to the study. For example, how many of the ten participants had regular contact with children? Were they parents, relatives (e.g., aunts or uncles), educators, or professionals working with children? Given their influence on the development of the project, this information appears relevant.**

Response: We agree that this information is relevant. Additional information on the interviewed participants, including their professional background and contact with children, is available in the study materials on the OSF. To improve transparency, we have added a reference to these materials in the manuscript:

“10 adult laypeople (60% female) with a mean age of 29.70 years (*SD* = 2.36) participated in individual semi-structured interviews designed to elicit beliefs about creativity in children and adults after giving verbal informed consent. Additional information on participant characteristics and study materials is available in the OSF repository.”

**6. Authors described that adult creativity was often characterized as more product- and goal-oriented, and situated within specific domains such as profession or hobbies. Did the authors collect the professional background of the adults in the main study? This variable may be important, as individuals who regularly engage in creative activities have been shown to outperform the general population in creativity-related tasks (e.g., Rondini et al., “Stable Diffusion Models Reveal a Persisting Human–AI Gap in Visual Creativity”, Advanced Science, 2026). If such information was not collected, the authors should acknowledge this as a potential limitation of the study.**

Response: Thank you for this comment. We did not collect detailed information on participants’ professional background or engagement in creative professions. While the adult sample was recruited from the general population and was not specifically selected based on creative expertise, we agree that individual difference in creative expression may influence performance on creativity tasks. We have therefore acknowledged this point as a limitation in the revised manuscript:

“Fifth, creative products were rated by adults; children might evaluate children’s products differently. In addition, we did not assess participants’ professional background or creative expertise, which may represent an additional source of variability in creative performance.”

**7. In the Introduction, the authors may wish additional constrained creative tasks involving drawing production, such as the Test of Creative Imagery Activities (TCIA; Jankowska & Karwowski, “Measuring Creative Imagery Abilities”, Frontiers in Psychology, 2015).**

Response: Thank you for this suggestion. We have added the TCIA as an additional example in the introduction.

**8. The assessment of the creative performance is a strength of the study, particularly the use of multiple raters and separate evaluations of novelty and effectiveness. However, did the authors examine the extent to which these two dimensions explain the overall creativity score? In other words, should overall creativity be interpreted as a distinct construct, or primarily as a combination of novelty and effectiveness?**

Response: Thank you for this insightful comment. To further examine the relationship between novelty, effectiveness, and overall creativity, we calculated response-level correlations for each task and report them in the Supplementary Materials (see Table S2). We also added a reference to these analyses in the *Scoring of creative performance* section. Overall creativity was strongly associated with novelty across all tasks, whereas associations with effectiveness varied substantially by task. These findings suggest that overall creativity judgments were closely linked to novelty, but that effectiveness ratings offered a complementary view on creative quality especially in the AUT.

“Interrater reliability was high across tasks and dimensions, with Cronbach’s α ranging from .789 to .980. Full reliability coefficients, including 95% confidence intervals, are reported in the Supplemental Analyses (see Table S1). Response-level correlations among novelty, effectiveness, and overall creativity ratings replicated that creativity evaluations are more strongly driven by the perceived novelty than effectiveness; detailed analyses are reported in the Supplemental Analyses (see Table S2).”

**S2 Table. Response-level correlations among novelty, effectiveness, and overall creativity ratings across creativity tasks.** * *p* < .05. ** *p* < .01. *** *p* < .001.

| Task | *r* Novelty, Effectiveness | *r* Novelty, Creativity | *r* Effectiveness, Creativity |
| --- | --- | --- | --- |
| Alternate Uses Task | -.09* | .92*** | .12** |
| Structured Creative Drawing | .90*** | .96*** | .96*** |
| Free Drawing | .39*** | .78*** | .83*** |

**9. It is not entirely clear who administered the task to the preschool children. Experimenter familiarity may influence children’s performance and should therefore be reported.**

Response: All participants were assessed individually by one of the authors, who was not familiar with the participants prior to testing. We have clarified this in the manuscript:

“All participants were tested individually between August 1, 2021, and April 30, 2022, by one of the authors, who was not familiar with the participants prior to testing.”

**10. Why was the feasibility of the procedure initially tested by two children aged four and seven years? Since the seven year-old is no longer a preschool child, some justification for this choice would be helpful.**

Response: The pilot testing was conducted to evaluate the comprehensibility and feasibility of the procedure and task instructions before the main study. At this stage, children from a somewhat broader age range were included to ensure that the tasks could be understood and completed appropriately across different developmental levels. We have clarified this rationale in the manuscript:

“The procedure was initially pilot-tested with two children (four and seven years old) to evaluate the comprehensibility and feasibility of the tasks and instructions across different developmental levels; the results informed final task adjustments.”

**11. The manuscript would benefit from the inclusion of example drawings. A figure showing representative examples from the different tasks and groups would be highly informative for readers.**

Response: We agree that example drawings can help readers better understand the nature of the drawing tasks and the types of responses obtained. While publication permission was not available for all participants, a subset of participants explicitly consented to the use of their drawings for publication purposes. We therefore added a selection of representative example drawings from these participants to the online supplementary materials and included a reference to these materials in the manuscript.

**12. The authors indicate that the five raters were blind to participant group. However, it seems plausible that group membership could often be inferred from the drawings themselves. For example, preschool children may use fewer colors, produce less complete human figures, or display other age-related characteristics. This potential limitation should be acknowledged.**

Response: Thank you for this important observation. Although raters were not informed about participants’ age group, we agree that age-related characteristics of the drawings may have allowed group membership to be inferred in some cases. We have acknowledged this as a limitation in the revised manuscript:

“In addition, we did not assess participants’ professional background or creative expertise, which may represent an additional source of variability in creative performance. Moreover, although raters were blind to participants’ age group, age-related characteristics of the drawings may have allowed group membership to be inferred in some cases.”

**13. In Figure 2, please explicitly indicate that the measure of general creative engagement was reported by the parents.**

Response: We have clarified this in the figure note:

**“Fig 2. General creative engagement, creative self-concept, and beliefs about creativity.** For preschool children, ratings of general creative engagement were provided by parents, whereas all other measures were self-reported.”

**14. Another strength of this study is the simultaneous evaluation of both the creative performance and the experience. To highlight this contribution, I suggest splitting the Figure 3 into two separate figures: one focusing on performance measures (Novelty, Effectiveness, and Creativity), and the other one on the experiential measures (Self-Efficacy, Motivation, Enjoyment, Self-Appraisal and Anxiety).**

Response: Thank you for this helpful suggestion. To better distinguish between creative performance and creative experience, we reorganized Figure 3 and added separate section headings for both domains.

**Fig 3. Creative task performance and subjective experience in the three creativity tasks for children and adults.** Asterisks indicate statistical significance of between-group comparisons with t-tests. *** *p* < .001.

**15. Considering the results across the three tasks, it is somewhat surprising that the novelty scores were lower in the free-drawing task for both groups. Could this reflect a limitation of the rating procedure, or perhaps the greater freedom of the task led participants to generate many ideas without fully developing a particularly original one? This pattern is interesting and may deserve consideration in the Discussion.**

Response: Thank you for this interesting observation. We agree that the pattern deserves consideration. We believe that the lower novelty ratings in the free-drawing task are likely attributable to the task instructions. Whereas the structured drawing task explicitly encouraged participants to create a novel fantasy animal, the free-drawing task allowed participants to draw any subject, including highly conventional and familiar objects. We have clarified this point in the manuscript:

“Adults showed consistently higher task performance than the preschool children in the AUT and the structured drawing task, as their ideas and drawings were rated as more novel, effective, and creative. In the free drawing task, adults also produced more effective and creative drawings than children, but groups did not differ in terms of novelty as adults often chose similarly common drawing topics. This pattern is consistent with the task instructions, as only the structured drawing task explicitly encouraged the production of a creative response.”

**16. The description of tasks’ duration should be complemented with quantitative data. Mean durations, standard deviations, and relevant statistical analyses comparing groups should be reported within the manuscript.**

Response: We agree that the description of task duration should be accompanied by quantitative information. We have therefore added the corresponding descriptive statistics to the manuscript:

“Interestingly, children spent less time than adults on the timed AUT [*M* = 1.92, *SD* = 0.87 vs. *M* = 2.72, *SD* = 0.61; *t*(159) = -6.46, *p* < .001] and the structured drawing task [*M* = 2.71, *SD* = 2.32 vs. *M* = 6.58, *SD* = 1.92; *t*(159) = -11.63, *p* < .001]; in the unstructured drawing task, which was untimed, children (*M* = 5.06, *SD* = 4.66) and adults (*M* = 4.16, *SD* = 2.90) spent a similar amount of time [*t*(154) = 1.50, *p* = .134].”
