# Supplementary material for: Are children really more creative than adults? A mixed-methods examination of creative performance and experience
Source: PLoS One. 2026 Oct 21;21(10):e0346553. doi: 10.1371/journal.pone.0346553 (PMC13686510; doi:10.1371/journal.pone.0346553)
Supplement: Peer review history

# Peer review: Decision Letter 0

**DOI:** 10.1371/journal.pone.0346553.r001

**Authors:** Henri Tilga

9 Apr 2026

PONE-D-26-13778

Are children really more creative than adults? A mixed-methods examination of creative performance and experience

PLOS One?

Dear Dr. Benedek,

Thank you for submitting your manuscript to PLOS ONE. After careful consideration, we feel that it has merit but does not fully meet PLOS ONE’s publication criteria as it currently stands. Therefore, we invite you to submit a revised version of the manuscript that addresses the points raised during the review process.

Please submit your revised manuscript by May 24 2026 11:59PM. If you will need more time than this to complete your revisions, please reply to this message or contact the journal office at plosone@plos.org. When you're ready to submit your revision, log on to https://www.editorialmanager.com/pone/ and select the 'Submissions Needing Revision' folder to locate your manuscript file.

- A letter that responds to each point raised by the academic editor and reviewer(s). You should upload this letter as a separate file labeled 'Response to Reviewers'.
- A marked-up copy of your manuscript that highlights changes made to the original version. You should upload this as a separate file labeled 'Revised Manuscript with Track Changes'.
- An unmarked version of your revised paper without tracked changes. You should upload this as a separate file labeled 'Manuscript'.

If you would like to make changes to your financial disclosure, please include your updated statement in your cover letter. Guidelines for resubmitting your figure files are available below the reviewer comments at the end of this letter.

If applicable, we recommend that you deposit your laboratory protocols in protocols.io to enhance the reproducibility of your results. Protocols.io assigns your protocol its own identifier (DOI) so that it can be cited independently in the future. For instructions see: https://journals.plos.org/plosone/s/submission-guidelines#loc-laboratory-protocols. Additionally, PLOS ONE offers an option for publishing peer-reviewed Lab Protocol articles, which describe protocols hosted on protocols.io. Read more information on sharing protocols at https://plos.org/protocols?utm_medium=editorial-email&utm_source=authorletters&utm_campaign=protocols.

As the corresponding author, your ORCID iD is verified in the submission system and will appear in the published article. PLOS supports the use of ORCID, and we encourage all coauthors to register for an ORCID iD and use it as well. Please encourage your coauthors to verify their ORCID iD within the submission system before final acceptance, as unverified ORCID iDs will not appear in the published article. *Only*  the individual author can complete the verification step; PLOS staff *cannot*  verify ORCID iDs on behalf of authors.

We look forward to receiving your revised manuscript.

Kind regards,

Henri Tilga, PhD

Academic Editor

PLOS One

Journal Requirements:

When submitting your revision, we need you to address these additional requirements.

1. Please ensure that your manuscript meets PLOS ONE's style requirements, including those for file naming. The PLOS ONE style templates can be found at

https://journals.plos.org/plosone/s/file?id=wjVg/PLOSOne_formatting_sample_main_body.pdf and https://journals.plos.org/plosone/s/file?id=ba62/PLOSOne_formatting_sample_title_authors_affiliations.pdf

2. Please include a complete copy of PLOS’ questionnaire on inclusivity in global research in your revised manuscript. Our policy for research in this area aims to improve transparency in the reporting of research performed outside of researchers’ own country or community. The policy applies to researchers who have travelled to a different country to conduct research, research with Indigenous populations or their lands, and research on cultural artefacts. The questionnaire can also be requested at the journal’s discretion for any other submissions, even if these conditions are not met.  Please find more information on the policy and a link to download a blank copy of the questionnaire here: https://journals.plos.org/plosone/s/best-practices-in-research-reporting. Please upload a completed version of your questionnaire as Supporting Information when you resubmit your manuscript.

3. Thank you for stating the following in the Acknowledgments Section of your manuscript:

We gratefully acknowledge the help of Rosalie Andrae, Luca Bartlmä, Felix Braun, Noah Meinzer, Eva Schmid, and Stefanie Schranz with creativity ratings and evaluations of the qualitative data. This research was funded in whole, or in part, by the Austrian Science Fund (FWF) (10.55776/P35661). For the purpose of open access, the author has applied a CC BY public copyright license to any Author Accepted Manuscript version arising from this submission.

We note that you have provided funding information that is not currently declared in your Funding Statement. However, funding information should not appear in the Acknowledgments section or other areas of your manuscript. We will only publish funding information present in the Funding Statement section of the online submission form.

Please remove any funding-related text from the manuscript and let us know how you would like to update your Funding Statement. Currently, your Funding Statement reads as follows:

MB received an award by the Austrian Science Fund (FWF; 10.55776/P35661; https://www.fwf.ac.at/).

The funders played no role in the study design, data collection and analysis, decision to publish, or preparation of the manuscript.

Please include your amended statements within your cover letter; we will change the online submission form on your behalf.

4. Please amend either the title on the online submission form (via Edit Submission) or the title in the manuscript so that they are identical.

5. Please upload a copy of Supporting Information Table S2, S3 which you refer to in your text on page 8, 10.

6.  Please include captions for your Supporting Information files at the end of your manuscript, and update any in-text citations to match accordingly. Please see our Supporting Information guidelines for more information: http://journals.plos.org/plosone/s/supporting-information.

7. If the reviewer comments include a recommendation to cite specific previously published works, please review and evaluate these publications to determine whether they are relevant and should be cited. There is no requirement to cite these works unless the editor has indicated otherwise.

[Note: HTML markup is below. Please do not edit.]

Reviewers' comments:

Reviewer's Responses to Questions

**Comments to the Author**

1. Is the manuscript technically sound, and do the data support the conclusions?

Reviewer #1: Yes

Reviewer #2: Partly

**********

2. Has the statistical analysis been performed appropriately and rigorously?

Reviewer #1: Yes

Reviewer #2: Yes

**********

3. Have the authors made all data underlying the findings in their manuscript fully available?

The PLOS Data policy

Reviewer #1: Yes

Reviewer #2: Yes

**********

4. Is the manuscript presented in an intelligible fashion and written in standard English?

Reviewer #1: Yes

Reviewer #2: Yes

**********

Reviewer #1: I would like to thank for the opportunity to review this manuscript. Please see the following comments to consider to further increase the quality of this manuscript.

The manuscript entitled “Are children really more creative than adults? A mixed-methods examination of creative performance and experience” addresses a timely and conceptually important question by integrating qualitative and quantitative approaches. The study is clearly structured, theoretically grounded, and methodologically transparent, and it makes a valuable contribution by disentangling creative performance from experiential and motivational aspects of creativity. The combination of a qualitative pre-study with an experimental comparison between preschool children and adults is particularly commendable, as it allows the authors to bridge lay beliefs and empirical evidence in a coherent manner. At the same time, several aspects of the manuscript could be strengthened to enhance its theoretical precision, methodological rigor, and overall clarity.

The introduction is generally well-written and situates the study within relevant literature; however, the theoretical framing could be sharpened further. Please consider clarifying more explicitly what is meant by “creativity” at different points in the manuscript, as the argument currently moves between divergent thinking, creative potential, creative behavior, and subjective experience without always clearly distinguishing these constructs. Although the authors acknowledge the multidimensionality of creativity, it would be beneficial to articulate a more explicit conceptual model early in the introduction that specifies how cognitive performance, affective-motivational experience, and self-beliefs are theoretically related. This would help readers better understand how the different measures used in the study map onto the broader construct of creativity. Perhaps the authors could also engage more critically with alternative frameworks, such as the distinction between “little-c” and “Big-C” creativity or the 4C model, to further situate their contribution.

The rationale for the qualitative pre-study is convincing, yet its integration into the overall research design could be elaborated. While the manuscript states that the qualitative findings informed the design of the main study, the exact pathways of this influence remain somewhat implicit. Please consider providing more concrete examples of how specific themes identified in the interviews translated into design choices, such as the selection of tasks or the inclusion of particular self-report measures. Additionally, the qualitative sample is relatively small (N = 10), which is not uncommon but nevertheless limits the breadth of perspectives captured. It would be helpful to acknowledge this more explicitly and perhaps justify why this sample size was considered sufficient for the purposes of the study.

The methodology of the quantitative study is generally robust and well-described, with clear procedures and open science practices (e.g., OSF repository) that are highly commendable . However, several methodological considerations merit further attention. First, the use of a fixed task order may introduce order or fatigue effects, particularly given the age differences between groups. Please consider discussing this limitation more explicitly and, if possible, justifying why counterbalancing was not implemented. Second, while the adaptation of tasks for children is appropriate, the comparability of measures across age groups remains a potential concern. For instance, the reliance on self-report measures in young children may raise questions about validity, as also briefly acknowledged in the discussion. It might strengthen the manuscript to elaborate on how these measures were validated or adapted for this age group and to what extent measurement invariance can be assumed.

The scoring of creative performance is a strength of the study, particularly the use of multiple raters and separate ratings of novelty and effectiveness. Nevertheless, please consider providing more detail on the training of raters and the rating procedure, as this would enhance transparency and reproducibility. In addition, the choice of the “max-3” scoring approach for the AUT is justified by prior work, but it might be useful to briefly explain its implications for interpretation, especially for readers less familiar with divergent thinking methodology.

The statistical analysis is appropriate overall, but some aspects could be clarified. The manuscript reports a stringent significance threshold (p < .001), which is commendable in terms of controlling Type I error, yet it would be helpful to provide a clearer rationale for this decision and to discuss its implications for statistical power. Furthermore, while effect sizes are reported, their interpretation could be expanded in the results section to aid readers in understanding the practical significance of the findings. Perhaps the authors could also consider complementing the frequentist analyses with confidence intervals more consistently in the main text, not only in the supplementary materials.

The results are clearly presented and logically organized, and the figures (e.g., Figures 2 and 3) effectively illustrate group differences across tasks and measures (see pages 27–28) . However, the narrative could be made slightly more concise by reducing some repetition between text and figures. At the same time, it might be beneficial to highlight key interaction effects more explicitly, particularly regarding how task structure moderates differences between children and adults.

The discussion provides a thoughtful interpretation of the findings and successfully reconciles the apparent discrepancy between lay beliefs and empirical evidence. The distinction between creative performance and creative engagement is a particularly valuable contribution. Nevertheless, some interpretations could be tempered to avoid overgeneralization. For example, the conclusion that children’s advantage lies in “creative agency” is plausible but somewhat speculative, given that agency was not directly measured. Please consider framing such claims more cautiously and grounding them more explicitly in the data.

The limitations section is appropriate and acknowledges several important issues, yet it could be expanded further. In particular, the cross-sectional design limits developmental inferences, and this could be discussed in more depth. Additionally, cultural factors may influence both lay beliefs and creative expression, and it might be worthwhile to note that the sample is drawn from a specific cultural context, which may limit generalizability.

The manuscript could be further strengthened by more explicitly integrating a self-determination theory (SDT) perspective to interpret the observed differences in creative engagement and experience. In particular, Ahmadi et al. (2023) provide a comprehensive and systematically developed classification of motivational behaviors that support or thwart the basic psychological needs of autonomy, competence, and relatedness, thereby shaping individuals’ motivation and engagement. Please consider using this framework to more precisely conceptualize children’s higher motivation, enjoyment, and confidence as indicators of need-supportive contexts, and adults’ comparatively higher anxiety as potentially linked to need-thwarting conditions. Such integration could offer a more theoretically grounded explanation of why children appear more “creative” in terms of engagement, and perhaps clarify how contextual factors and task structures influence creative expression across age groups.

Ahmadi, A., Noetel, M., Parker, P., Ryan, R. M., Ntoumanis, N., Reeve, J., Beauchamp, M., Dicke, T., Yeung, A., Ahmadi, M., Bartholomew, K., Chiu, T. K. F., Curran, T., Erturan, G., Flunger, B., Frederick, C., Froiland, J. M., González-Cutre, D., Haerens, L., . . . Lonsdale, C. (2023). A classification system for teachers’ motivational behaviors recommended in self-determination theory interventions. Journal of Educational Psychology, 115(8), 1158–1176. https://doi.org/10.1037/edu0000783

Reviewer #2: This study by Saretzki et al. investigates different aspects of creativity in preschool children and adults.

It is an interesting study, but many clarifications are needed. The main weakness is the lack of information on participants and participant selection, which limits interpretations, in my opinion. I detailed my concerns below. But I really appreciate that all data and questionnaires are open-source.

1- Please provide references for the statement, lines 34-37: “At the same time, this believe appears to contrast…”.

2- For the qualitative study, I am unsure how much this brings valid results, as the sample of 10 individuals is really low. Further, no information is provided on how these 10 people got selected. Given the small sample, any interpretations can be easily biased based on potential disorders, age, culture, education level and, maybe more importantly, if they have children (I believe that could make a big difference in overall beliefs about children’s creativity). I think, my main question is why should I trust their opinion to know anything about children’s creativity? Any discussion / results related to this qualitative study should be more cautious in their validity and suggest that these results should not be generalized.

3- While everything is provided on OSF, more details on the questionnaires should still be provided in the method as the paper should stand on its own.

4- For the quantitative study, inclusion / exclusion criteria should be provided for the participants. What about neurodevelopmental disorders? Where the participants all considered healthy / typically developing? If disorders and developmental delays were not considered (especially in the children, but it also matters in the adult group), it is a major limitation as it is likely that ADHD or autism may impact the level of creativity or attention needed to complete the tasks. How did these participants get recruited? Without this information, I don’t believe that results can be fully interpreted.

5- Related to my previous comment, my other major concern is how did the investigators ensure that the children understood all the questions and the concept of being anxious / worried, or the concept of “creativity”, especially in the youngest children (Some were only 4!), or in children that may have developmental delay (if this was not recorded). It is therefore possible that the group differences noted were not due to development per se, but due to disorders, lack of understanding, lack of being aware of their own anxiety (because they are too young to understand it), etc.

6- The age range of the adults recruited was rather large (30 years). As for developmental effects of age on creativity, it is known that aging is associated with changes in creativity. It does not seem that there was any consideration for potential age effect within the adult groups. It could cause increased variability and reduce effect sizes.

7- Please provide a reference for the AUT.

8- Given the very restricted age range of the children included in the study, I think the discussion should be more precise and not generalized to “children and adults” as it is likely going to change if they had 10 years old. While this is acknowledged in the limitation, it should be more precise through the discussion (e.g., “preschool children” vs “children”). Otherwise, I think the interpretations are over-stating the findings.

**********

what does this mean?). If published, this will include your full peer review and any attached files.

If you choose “no”, your identity will remain anonymous but your review may still be made public.

**Do you want your identity to be public for this peer review?**  For information about this choice, including consent withdrawal, please see our Privacy Policy

Reviewer #1: No

Reviewer #2: No

**********

[NOTE: If reviewer comments were submitted as an attachment file, they will be attached to this email and accessible via the submission site. Please log into your account, locate the manuscript record, and check for the action link "View Attachments". If this link does not appear, there are no attachment files.]

To ensure your figures meet our technical requirements, please review our figure guidelines: https://journals.plos.org/plosone/s/figures

You may also use PLOS’s free figure tool, NAAS, to help you prepare publication quality figures: https://journals.plos.org/plosone/s/figures#loc-tools-for-figure-preparation.

NAAS will assess whether your figures meet our technical requirements by comparing each figure against our figure specifications.

# Author comment: Author response to Decision Letter 1

**DOI:** 10.1371/journal.pone.0346553.r002

30 Apr 2026

Dear Editor, dear Reviewers,

Thank you very much for reviewing our manuscript entitled Are children really more creative than adults? A mixed-methods examination of creative performance and experience and for your thorough examination of the manuscript and for providing constructive and insightful feedback on how to improve it. Please find below a detailed description of how each Reviewer point was addressed in the revised manuscript. We believe that careful consideration of the thoughtful feedback has helped to strengthen the article substantially.

In addition, we carefully revised the manuscript to ensure full compliance with the journal’s formatting and reporting requirements.

Sincerely,

The Authors

Reviewer #1

I would like to thank for the opportunity to review this manuscript. Please see the following comments to consider to further increase the quality of this manuscript.

The manuscript entitled “Are children really more creative than adults? A mixed-methods examination of creative performance and experience” addresses a timely and conceptually important question by integrating qualitative and quantitative approaches. The study is clearly structured, theoretically grounded, and methodologically transparent, and it makes a valuable contribution by disentangling creative performance from experiential and motivational aspects of creativity. The combination of a qualitative pre-study with an experimental comparison between preschool children and adults is particularly commendable, as it allows the authors to bridge lay beliefs and empirical evidence in a coherent manner.

At the same time, several aspects of the manuscript could be strengthened to enhance its theoretical precision, methodological rigor, and overall clarity.

The introduction is generally well-written and situates the study within relevant literature; however, the theoretical framing could be sharpened further. Please consider clarifying more explicitly what is meant by “creativity” at different points in the manuscript, as the argument currently moves between divergent thinking, creative potential, creative behavior, and subjective experience without always clearly distinguishing these constructs. Although the authors acknowledge the multidimensionality of creativity, it would be beneficial to articulate a more explicit conceptual model early in the introduction that specifies how cognitive performance, affective-motivational experience, and self-beliefs are theoretically related. This would help readers better understand how the different measures used in the study map onto the broader construct of creativity.

Perhaps the authors could also engage more critically with alternative frameworks, such as the distinction between “little-c” and “Big-C” creativity or the 4C model, to further situate their contribution.

Response: We agree that more background on the diverse facets of creativity and their interplay is helpful to better contextualize the measures of this study. We revised and extended the relevant section in the literature accordingly:

“Creativity is a multifaceted construct that extends beyond creative thinking ability to include relevant traits, skills, and behavioral components. Specifically, creative behavior and achievement is known to develop and arise from the interplay of cognitive abilities (e.g., DT ability), core personality traits (e.g., openness to new experiences), creative self-beliefs (e.g., creative confidence) and expertise in the context of environmental influences [15,16]. Some of these facets, such as domain-specific expertise and lifetime creative achievement, depend on accumulated knowledge and prolonged practice and therefore typically increase with age, favoring adults [17]. For example, professional or eminent levels of creativity (i.e., Pro-c and Big-C) are typically observed only at later career stages or even assigned posthumous [18,19]. Other facets may align more closely with popular intuitions about children’s creativity. Children may engage with creative tasks in a more play-like manner [20,21] reflecting high openness [22], and intrinsic motivation [23], thus enjoying the task without being overly concerned about the outcome [24]. Adults, in contrast, are more aware of their strengths and weaknesses [25] and typically more sensitive to external expectations and social evaluation [26], which may support goal-directed performance via metacognition and self-regulation [27,28] but also increase worries and constrain risk-taking [29,30]. Considering both cognitive and non-cognitive aspects of creativity therefore appears essential when examining whether children are more or less creative than adults.”

The rationale for the qualitative pre-study is convincing, yet its integration into the overall research design could be elaborated. While the manuscript states that the qualitative findings informed the design of the main study, the exact pathways of this influence remain somewhat implicit. Please consider providing more concrete examples of how specific themes identified in the interviews translated into design choices, such as the selection of tasks or the inclusion of particular self-report measures.

Response: While the manuscript already indicated that the qualitative pre-study informed the design of the main study, we agree that this link can be made more explicit. We therefore revised and expanded the section on design implications accordingly:

“Design implications for the main study

Qualitative findings suggested that children’s creativity is commonly associated with openness, spontaneity, intrinsic motivation, and freedom from external constraints, whereas adults’ creativity was primarily associated with goal orientation, accumulated knowledge and experience, and domain specificity. Concrete expressive behaviors (e.g., drawing, storytelling, experimenting) were mentioned for both groups but contextualized differently: children’s creativity was typically described in terms of playful exploration, spontaneous experimentation, and low inhibitions, whereas adults’ creativity was more often characterized as more product- and goal-oriented, and situated within specific domains such as profession or hobbies. These insights motivated us to examine creative performance under two conditions that might favor children versus adults [20, but see 34], respectively: 1) an unconstrained, play-like condition that does pose a clear goal or time-limit (cf. free drawing task), and 2) a constrained, test-like condition that has a clear goal and time-limit (cf. structured drawing task). Additionally, we included the well-established alternative uses task for reference with the rich literature using this task [35] and scored creative performance independently for novelty, effectiveness and overall creativity [36], as children’s playful exploration behavior might yield more novel yet less effective responses.

Environmental references centered on freedom, with children frequently described as having fewer external constraints and greater latitude for exploration, while adults were portrayed as facing time pressure, social expectations, and performance demands. Therefore, we decided to measure subjective experience during the creative tasks besides actual task performance. Finally, since participants predominantly located peak creativity in early childhood, particularly shortly before school entry, we decided to focus on preschool children in the comparison with adults.”

Additionally, the qualitative sample is relatively small (N = 10), which is not uncommon but nevertheless limits the breadth of perspectives captured. It would be helpful to acknowledge this more explicitly and perhaps justify why this sample size was considered sufficient for the purposes of the study.

Response: We agree that the qualitative sample size may seem relatively small and appreciate the opportunity to clarify this point. While a sample size of N = 10 limits the breadth of perspectives captured, it is consistent with methodological recommendations for achieving thematic saturation in interview-based research. Recent reviews suggest that saturation is often reached within approximately 9-17 interviews (Hennink & Kaiser, 2022) or even around 9 interviews for theme-based analyses (Wutich et al., 2024).

Importantly, the aim of the qualitative pre-study was not to provide an exhaustive account of lay beliefs, but rather to identify central and recurring themes that could inform the design of the subsequent quantitative study. In this context, the sample size was considered sufficient to capture the most salient intuitions relevant to our research aims.

We have now made this rationale more explicit in the manuscript:

“10 adult laypeople (60% female) with a mean age of 29.70 years (SD = 2.36) participated in semi-structured interviews designed to elicit beliefs about creativity in children and adults after giving verbal informed consent. Participants were recruited via convenience sampling from the local community and the researchers’ personal networks. Interviews addressed perceived developmental changes in creativity (including the age at which children are considered the most creative) and beliefs about characteristics and behaviors associated with creativity in children versus adults. Interviews were audio-recorded, transcribed verbatim, and analyzed using qualitative content analysis. The sample size was consistent with recommendations for achieving thematic saturation in interview-based research [31,32] and appropriate for the exploratory aim of the pre-study.”

Hennink, M., & Kaiser, B. N. (2022). Sample sizes for saturation in qualitative research: A systematic review of empirical tests. Social science & medicine, 292, 114523. https://doi.org/10.1016/j.socscimed.2021.114523

Wutich, A., Beresford, M., & Bernard, H. R. (2024). Sample sizes for 10 types of qualitative data analysis: an integrative review, empirical guidance, and next steps. International Journal of Qualitative Methods, 23, 16094069241296206. https://doi.org/10.1177/16094069241296206

The methodology of the quantitative study is generally robust and well-described, with clear procedures and open science practices (e.g., OSF repository) that are highly commendable. However, several methodological considerations merit further attention.

First, the use of a fixed task order may introduce order or fatigue effects, particularly given the age differences between groups. Please consider discussing this limitation more explicitly and, if possible, justifying why counterbalancing was not implemented.

Response: The free drawing task preceded the structured drawing task because it was framed as a familiarization with the task materials. The AUT could have been counterbalanced relative to the drawing tasks, but we always kept it first as we did not plan to compare performance between these tasks, but we agree that this approach does not control for carry-over and time effects. We now discuss this as a limitation:

“Fourth, other creativity tasks (e.g., insight problem-solving) could be included and task order should be counterbalanced to limit fatigue effects.”

Second, while the adaptation of tasks for children is appropriate, the comparability of measures across age groups remains a potential concern. For instance, the reliance on self-report measures in young children may raise questions about validity, as also briefly acknowledged in the discussion. It might strengthen the manuscript to elaborate on how these measures were validated or adapted for this age group and to what extent measurement invariance can be assumed.

Response: We now provide more information on how measures were adapted to the young target group, informed by cognitive surveys with two young girls.

“The feasibility of the procedure was initially tested in a cognitive survey involving two children (four and seven years old), which informed final task adjustments. “

“To account for the children’s limited experience with the object, an actual tin can was provided to all participants (children and adults), allowing them to explore it during the task.”

“Following similar assessments with young children, we used brief items that were communicated orally in an age-appropriate language using a five-point scale anchored with emoticons [e.g., 38]. For each item, all response options were described by the experimenter, and the question was rephrased if necessary.”

The performance measures were found to work well as responses were task-relevant (e.g., the structured drawing task resulted in recognizable fantasy animals). The single item measures prevented us from running proper analyses of measurement invariance. For self-report questions, children sometimes required a rephrasing of the question. Despite all measures of precaution, we cannot rule out that preschool children have a different understanding of the constructs and differ in their general use of the response scale. Therefore, we discuss these important points as study limitations.

The scoring of creative performance is a strength of the study, particularly the use of multiple raters and separate ratings of novelty and effectiveness. Nevertheless, please consider providing more detail on the training of raters and the rating procedure, as this would enhance transparency and reproducibility.

Response: We have now clarified the rater training and rating procedure in the Methods section. Specifically, we added that the initial training involved familiarization with the rating scales and criteria, followed by supervised practice on a subset of responses and drawings, including discussion of uncertainties, before the remaining responses were evaluated independently.

In addition, the choice of the “max-3” scoring approach for the AUT is justified by prior work, but it might be useful to briefly explain its implications for interpretation, especially for readers less familiar with divergent thinking methodology.

Response: We agree that a clarification of the rationale for using the max-3 scoring approach is warranted. We have therefore expanded the description in the Methods section to clarify that this approached reduced confounding effects of ideational fluency by focusing on the quality of participants’ best three ideas. Specifically, max-3 scores are based on the average ratings of the three highest-rated responses per participant:

“For the AUT, a max-3 aggregation approach was applied to reduce response fluency confounds by focusing on a fixed number of responses; specifically, scores reflect the average ratings of the three highest-rated responses per participant [39].”

The statistical analysis is appropriate overall, but some aspects could be clarified. The manuscript reports a stringent significance threshold (p < .001), which is commendable in terms of controlling Type I error, yet it would be helpful to provide a clearer rationale for this decision and to discuss its implications for statistical power.

Response: We agree that the choice of a stringent significance threshold requires clarification. Given the number of statistical tests conducted, the use of a conservative threshold (p < .001) appears justified, as it approximates the level of stringency that would result from formal multiple comparison corrections (e.g., Bonferroni-type adjustments). Rather than applying varying corrections across analyses, we opted for a uniform threshold to ensure transparency and consistency. Such stringent thresholds have also been discussed in the literature to strengthen the evidential value of findings (e.g., Benjamin et al., 2018). We have clarified this rationale in the manuscript:

“All tests were two-tailed. To account for multiple comparisons, a conservative significance threshold of p < .001 was applied. This threshold approximated the level of stringency achieved by common correction procedures and ensures a consistent criterion across analyses.”

Benjamin, D. J., Berger, J. O., Johannesson, M., Nosek, B. A., Wagenmakers, E. J., Berk, R., ... & Johnson, V. E. (2018). Redefine statistical significance. Nature Human Behaviour, 2(1), 6-10. https://doi.org/10.1038/s41562-017-0189-z

Furthermore, while effect sizes are reported, their interpretation could be expanded in the results section to aid readers in understanding the practical significance of the findings. Perhaps the authors could also consider complementing the frequentist analyses with confidence intervals more consistently in the main text, not only in the supplementary materials.

Response: To improve the interpretation of our findings, we now report 95% confidence intervals alongside all key statistics in the main text, thereby facilitating a clearer understanding of their practical significance.

The results are clearly presented and logically organized, and the figures (e.g., Figures 2 and 3) effectively illustrate group differences across tasks and measures (see pages 27–28). However, the narrative could be made slightly more concise by reducing some repetition between text and figures. At the same time, it might be beneficial to highlight key interaction effects more explicitly, particularly regarding how task structure moderates differences between children and adults.

Response: We now discuss the significant interactions more explicitly:

“Significant interactions for all three performance measures indicated that smaller group differences were observed for novelty and creativity in the unstructured drawing, whereas group differences in effectiveness were highest in the structured drawing task.”

“Significant interactions for motivation and enjoyment indicated that adults generally felt more comfortable in the structured drawing task, whereas children enjoyed the free drawing task more.”

The discussion provides a thoughtful interpretation of the findings and successfully reconciles the apparent discrepancy between lay beliefs and empirical evidence. The distinction between creative performance and creative engagement is a particularly valuable contribution. Nevertheless, some interpretations could be tempered to avoid overgeneralization. For example, the conclusion that children’s advantage lies in “creative agency” is plausible but somewhat speculative, given that agency was not directly measured. Please consider framing such claims more cautiously and grounding them more explicitly in the data.

Response: We agree that the early reference to creative agency in the discussion came a bit out of the blue. We removed this early reference and introduce this notion more carefully with reference to the Creative Behavior as Agentic Action model by Karwowski and Beghetto (2019):

“Following the Creative Behavior as Agentic Action model [47], creative behavior is not just a function of ability, but also of creative confidence beliefs. In our study, children exhibited lower creative thinking potential than adults, yet higher creative confidence and overall higher creative agency, in terms of their intrinsic willingness to engage in creative behavior.”

The limitations section is appropriate and acknowledges several important issues, yet it could be expanded further. In particular, the cross-sectional design limits developmental inferences, and this could be discussed in more depth. Additionally, cultural factors may influence both lay beliefs and creative expression, and it might be worthwhile to note that the sample is drawn from a specific cultural context, which may limit generalizability.

Response: We fully agree that both the cross-sectional design and the cultural context of the sample limit the generalizability of the findings. We therefore revised the limitations section to acknowledge these points more explicitly:

“First, cross-sectional comparisons are susceptible to cohort effects and do not enable causal inference.”

“Finally, the findings are based on samples drawn from a specific cultural context. Future research could examine how lay beliefs and creative expression vary across different populations and backgrounds.”

The manuscript could be further strengthened by more explicitly integrating a self-determination theory (SDT) perspective to interpret the observed differences in creative engagement and experience. In particular, Ahmadi et al. (2023) provide a comprehensive and systematically developed classification of motivational behaviors that support or thwart the basic psychological needs of autonomy, competence, and relatedness, thereby shaping individuals’ motivation and engagement. Please consider using this framework to more precisely conceptualize children’s higher motivation, enjoyment, and confidence as indicators of need-supportive contexts, and adults’ comparatively higher anxiety as potentially linked to need-thwarting conditions. Such integration could offer a more theoretically grounded explanation of why children appear more “creative” in terms of engagement, and perhaps clarify how contextual factors and task structures influence creative expression across age groups.

Ahmadi, A., Noetel, M., Parker, P., Ryan, R. M., Ntoumanis, N., Reeve, J., Beauchamp, M., Dicke, T., Yeung, A., Ahmadi, M., Bartholomew, K., Chiu, T. K. F., Curran, T., Erturan, G., Flunger, B., Frederick, C., Froiland, J. M., González-Cutre, D., Haerens, L., … Lonsdale, C. (2023). A classification system for teachers’ motivational behaviors recommended in self-determination theory interventions. Journal of Educational Psychology, 115(8), 1158–1176. https://doi.org/10.1037/edu0000783

Response: We seized this suggestion and now added an interpretation of our findings in the context of the motivational behaviors in the context of SDT:

“This heightened agency in children can also be interpreted through the lens of Self-Determination Theory and relevant motivational behaviors [48]. Children’s higher enjoyment and confidence could be seen as indicators of need-supportive contexts, where the basic psychological needs for autonomy and competence are satisfied, likely because children perceive creative tasks as low-stakes, playful activities [49]. Conversely, the adults’ higher anxiety suggests need-thwarting conditions—where evaluative pressure or rigid self-expectations may undermine their autonomy and competence.”

Reviewer #2

This study by Saretzki et al. investigates different aspects of creativity in preschool children and adults. It is an interesting study, but many clarifications are needed. The main weakness is the lack of information on participants and participant selection, which limits interpretations, in my opinion. I detailed my concerns below. But I really appreciate that all data and questionnaires are open-source.

Please provide references for the statement, lines 34-37: “At the same time, this believe appears to contrast…”.

Response: We now support this statement with relevant references:

At the same time, this belief appears to contrast with evidence from age-normed creativity tests and meta-analytical work, which typically show a steady increase in creative thinking performance from childhood into adulthood [4–7].

For the qualitative study, I am unsure how much this brings valid results, as the sample of 10 individuals is really low. Further, no information is provided on how these 10 people got selected. Given the small sample, any interpretations can be easily biased based on potential disorders, age, culture, education level and, maybe more importantly, if they have children (I believe that could make a big difference in overall beliefs about children’s creativity). I think, my main question is why should I trust their opinion to know anything about children’s creativity? Any discussion/results related to this qualitative study should be more cautious in their validity and suggest that these results should not be generalized.

Response: We agree that the qualitative pre-study is based on a sample that may seem relatively small and non-representative and is therefore not intended to support generalizable conclusions. Consistent with our response to a similar comment by Reviewer 1, we now justify the sample size in more detailby noting that it aligns with methodological recommendations for interview-based research and with evidence suggesting that thematic saturation is often reached within approximately 9-17 interviews or even around 9 interviews for theme-based analyses (Hennink & Kaiser, 2022; Wutich et al., 2024).

Importantly, the aim of the pre-study was not to provide valid estimates of children’s creativity, but to integrate the perspective of lay people to inform the design of the quantitative main study. Participants were recruited via convenience sampling for this exploratory purpose. We have clarified the recruitment procedure in the Methods section and added a corresponding limitation emphasizing the restricted generalizability of the findings and their potential dependence on participants’ backgrounds.

While everything is provided on OSF, more details on the questionnaires should still be provided in the method as the paper should stand on its own.

Response: We agree that the manuscript should provide sufficient detail to understand the assessed constructs and procedures independently of supplemental materials. In the revised version, we have therefore added more details on creative thinking tasks and the self-report measures, including necessary adaptations for the young age group. For example, we describe that self-report measures used brief, age-appropriate items, oral presentations, and a five-point response scale anchored with emoticons that were individually described by the experimenter. We also provide the questions of single self-report items in the main text.

For the quantitative study, inclusion/exclusion criteria should be provided for the participants. What about neurodevelopmental disorders? Where the participants all considered healthy/typically developing? If disorders and developmental delays were not considered (especially in the children, but it also matters in the adult group), it is a major limitation as it is likely that ADHD or autism may impact the level of creativity or attention needed to complete the tasks. How did these participants get recruited? Without this information, I don’t believe that results can be fully interpreted.

Response: We agree that it is helpful to offer more information on the sample recruiting strategy. This study aimed for largely representative samples within the target age groups. A comprehensive developmental and clinical assessment for all participants was not feasible in the context of this study. Children with severe, clinical conditions would, however, typically not attend the public kindergarten. It should also be noted that the relationship between creativity and subclinical conditions were found to be subtle and complex, and not necessarily negative (e.g., Boot et al., 2017; Hoogman et al., 2020). In sum, we believe that it is unlikely that the consistent performance differences observed between children and adults are due to neurodevelopmental disorders. We now provide more information on the recruitment of participants:

“Children were recruited from nine local kindergartens in their final year prior to school entry. Adults were recruited from the general population. The study aimed for largely representative samples within preschoolers and adults and therefore did not apply other formal inclusion/exclusion criteria.”

Boot, N., Nevicka, B., & Baas, M. (2017). Subclinical symptoms of attention-deficit/hyperactivity disorder (ADHD) are associated with specific creative processes. Personality and Individual Differences, 114, 73–81. https://doi.org/10.1016/j.paid.2017.03.050

Hoogman, M., Stolte, M., Baas, M., & Kroesbergen, E. (2020). Creativity and ADHD: A review of behavioral studies, the effect of psychostimulants and neural underpinnings. Neuroscience & Biobehavioral Reviews, 119, 66-85. https://doi.org/10.1016/j.neubiorev.2020.09.029

Related to my previous comment, my other major concern is how did the investigators ensure that the children understood all the questions and the concept of being anxious/worried, or the concept of “creativity”, especially in the youngest children (Some were only 4!), or in children that may have developmental delay (if this was not recorded). It is therefore possible that the group differences noted were not due to development per se, but due to disorders, lack of understanding, lack of being aware of their own anxiety (because they are too young to understand it), etc.

Response: We agree that fair group comparisons rely on proper understanding of the tasks and scales, especially for the young age group. We now provide additional details on the measures taken to this end, including cognitive surveys, the use of age-appropriate language, oral instructions and responses and, if needed, additional explanations:

“To account for the children’s limited experience with the object, an actual tin can was provided to all participants (children and adults), allowing them to explore it during the task. Responses were given orally, recorded, and later transcribed, consistent with established procedures for assessing creativity in young children [5].”

“Following similar assessments with young children, we used brief items that were communicated orally in an age-appropriate language using a five-point scale anchored with emoticons [e.g., 38]. For each item, all response options were described by the experimenter, and the question was rephrased if necessary.”

The performance measures showed face validity as responses were task-relevant (e.g., the structured drawing task resulted in recognizable fantasy animals). For self-report questions, children sometimes required a rephrasing of the question. Despite all employed measures, we cannot rule out that preschool children have a different understanding of concepts like creativity or emotions and also differ in their general use of the response scale. Therefore, we discuss these points as important study limitations and advise that findings need to be interpreted cautiously in their context.

The age range of the adults recruited was rather large (30 years). As for developmental effects of age on creativity, it is known that aging is associated with changes in creativity. It does not seem that there was any consideration for potential age effect within the adult groups. It could cause increased variability and reduce effect sizes.

Response: Most of the adult participants were young adults (mean age = 25.31, SD = 5.90). To our knowledge, creativity is fairly stable in the young adult age range, and most pronounced developmental effects are observed either during childhood or late age. We now provide a more specific age description by clarifying for adults that “85% were between 20 and 30 years”.

Please provide a reference for the AUT.

Response: We note that the AUT is not a single standardized instrument with a definitive reference, but rather a widely used divergent thinking paradigm that varies in its administration and scoring across studies (see also Saretzki et al., 2026). To improve clarity and provide a conceptual anchor, we have now added a reference to Guilford (1967), who introduced such tasks in his seminal work on divergent thinking.

Given the very restricted age range of the children included in the study, I think the discussion should be more precise and not generalized to “children and adults” as it is likely going to change if they had 10 years old. While this is acknowledged in the limitation, it should be more precise through the discussion (e.g., “preschool children” vs “children”). Otherwise, I think the interpretations are over-stating the findings.

Response: We agree that the age range of the children sample should be reflected accurately. We have therefore revised relevant sections to refer specifically to preschool children rather than children in general. For example, methods and results clearly introduce the sample as preschool children but also use the shorter term “children” occasionally to avoid repetitive language. We thoroughly revised the discussion to specify that findings refer to preschool children and only retained the broader term “children” in places where it refers to common beliefs or prior literature. These revisions ensure that the scope of our conclusions is appropriately bounded and does not overgeneralize beyond the studied age group.

Attachment Submitted filename: Response to Reviewers.docx

# Peer review: Decision Letter 1

**DOI:** 10.1371/journal.pone.0346553.r003

**Authors:** Mohammad Salah Hassan

3 Jun 2026

-->Are children really more creative than adults?

Dear Dr. Benedek,

Please submit your revised manuscript by  Jul 18 2026 11:59PM. If you will need more time than this to complete your revisions, please reply to this message or contact the journal office at plosone@plos.org. When you're ready to submit your revision, log on to https://www.editorialmanager.com/pone/ and select the 'Submissions Needing Revision' folder to locate your manuscript file.

- A letter that responds to each point raised by the academic editor and reviewer(s). You should upload this letter as a separate file labeled 'Response to Reviewers'.
- A marked-up copy of your manuscript that highlights changes made to the original version. You should upload this as a separate file labeled 'Revised Manuscript with Track Changes'.
- An unmarked version of your revised paper without tracked changes. You should upload this as a separate file labeled 'Manuscript'.

If you would like to make changes to your financial disclosure, please include your updated statement in your cover letter. Guidelines for resubmitting your figure files are available below the reviewer comments at the end of this letter.

If applicable, we recommend that you deposit your laboratory protocols in protocols.io to enhance the reproducibility of your results. Protocols.io assigns your protocol its own identifier (DOI) so that it can be cited independently in the future. For instructions see: https://journals.plos.org/plosone/s/submission-guidelines#loc-laboratory-protocols. Additionally, PLOS ONE offers an option for publishing peer-reviewed Lab Protocol articles, which describe protocols hosted on protocols.io. Read more information on sharing protocols at https://plos.org/protocols?utm_medium=editorial-email&utm_source=authorletters&utm_campaign=protocols.

As the corresponding author, your ORCID iD is verified in the submission system and will appear in the published article. PLOS supports the use of ORCID, and we encourage all coauthors to register for an ORCID iD and use it as well. Please encourage your coauthors to verify their ORCID iD within the submission system before final acceptance, as unverified ORCID iDs will not appear in the published article. *Only*  the individual author can complete the verification step; PLOS staff *cannot*  verify ORCID iDs on behalf of authors.

We look forward to receiving your revised manuscript.

Kind regards,

Mohammad Salah Hassan, Ph.D

Academic Editor

PLOS One

Journal Requirements:

If the reviewer comments include a recommendation to cite specific previously published works, please review and evaluate these publications to determine whether they are relevant and should be cited. There is no requirement to cite these works unless the editor has indicated otherwise.

Additional Editor Comments:

Dear Authors,

Thank you for submitting the revised version of your manuscript entitled “Are children really more creative than adults? A mixed-methods examination of creative performance and experience” to PLOS ONE.

The revised manuscript has now been evaluated by the reviewers. I am pleased to note that the reviewers generally acknowledge the improvements made in response to the previous round of comments. In particular, two reviewers were satisfied with the revisions and expressed positive evaluations of the manuscript.

However, one reviewer has identified several remaining issues that require further attention before the manuscript can be considered for publication. These comments primarily relate to methodological clarification, reporting transparency, figure presentation, additional discussion of certain findings, and acknowledgment of study limitations. While these concerns do not appear to undermine the overall quality of the work, they should be carefully addressed to further strengthen the manuscript.

Therefore, I invite you to revise the manuscript and provide a detailed point-by-point response to all remaining reviewer comments. Please clearly indicate all changes made in the revised version.

I appreciate the effort invested in improving the manuscript and look forward to receiving your revised submission.

Sincerely,

Academic Editor

PLOS ONE

[Note: HTML markup is below. Please do not edit.]

Reviewers' comments:

Reviewer's Responses to Questions

**Comments to the Author**

Reviewer #2: All comments have been addressed

Reviewer #3: (No Response)

Reviewer #4: (No Response)

**********

2. Is the manuscript technically sound, and do the data support the conclusions?

Reviewer #2: Yes

Reviewer #3: Yes

Reviewer #4: Yes

**********

3. Has the statistical analysis been performed appropriately and rigorously?

Reviewer #2: Yes

Reviewer #3: Yes

Reviewer #4: Yes

**********

4. Have the authors made all data underlying the findings in their manuscript fully available?

The PLOS Data policy

Reviewer #2: Yes

Reviewer #3: Yes

Reviewer #4: Yes

**********

5. Is the manuscript presented in an intelligible fashion and written in standard English?

Reviewer #2: Yes

Reviewer #3: Yes

Reviewer #4: Yes

**********

Reviewer #2: (No Response)

Reviewer #3: Are children really more creative than adults? A mixed-methods examination of creative performance and experience

I found this to be a thoughtful and conceptually interesting paper that makes a worthwhile contribution to the creativity literature. In particular, I valued the authors’ attempt to disentangle creative performance from creative engagement, and to empirically examine why children are often perceived as “more creative” despite adults outperforming them on externally evaluated creative tasks. The overall pattern of findings was coherent and theoretically generative, especially the distinction between children’s stronger affective engagement (e.g., higher confidence, enjoyment, and intrinsic motivation) and adults’ stronger performance under evaluative or goal-directed conditions. I also appreciated the inclusion of lower-constraint tasks within the design, which strengthened the study by moving beyond purely structured creativity assessments. Overall, I found the paper clearly written, theoretically engaged, and addressing a genuinely interesting and culturally resonant question.

Additional reflections and future directions

One avenue that may be fruitful for future research concerns forms of creativity that are less product-oriented and less tightly tied to externally evaluated performance. While the inclusion of lower-constraint tasks strengthens the present design, many everyday perceptions of children’s creativity may emerge from more socially situated and play-based contexts, such as pretend play, collaborative imaginative activity, narrative improvisation, or spontaneous symbolic exploration. Investigating creativity in such settings may help further clarify the distinction observed here between creative performance and creative engagement/agency. For example, in genuinely open-ended collaborative imaginative contexts, adults may demonstrate advantages in sustaining coherent narratives, integrating abstraction or humour, and collectively scaffolding complex symbolic worlds, even while children appear more spontaneous, playful, or intrinsically immersed in the activity itself.

The findings also seem to create a natural opportunity for a qualitative follow-up study with the same adult participants. A particularly interesting feature of the present design is that the adult participants both completed the creativity tasks and, as a group, strongly endorsed the view that children are more creative. This places them in a unique position to help unpack the apparent disconnect between everyday beliefs about children’s creativity and the pattern of task-based performance reported in the study. For example, participants could be presented with the study’s findings and invited to reflect on whether the results surprised them, whether they viewed the administered tasks as representative of creativity more broadly, and whether their perceptions of children’s creativity are grounded more in observable engagement, spontaneity, playfulness, or imaginative expression than in externally evaluated creative products. Such a follow-up could provide particularly rich insight into some of the interpretive tensions the paper itself raises regarding the relationship between perceived creativity, creative engagement, and creative performance.

Reviewer #4: I would like to thank for the opportunity to review this manuscript entitled “Are children really more creative than adults? A mixed-methods examination of creative performance and experience”.

The study is well-structured and motivated, and it provides a valuable contribution to the creativity literature. Nevertheless, several aspects should be addressed to strengthen the manuscript and improve its overall impact.

1. As a general comment, the resolution of figures should be improved.

2. Based on the methods and terminology used throughout the manuscript, the title should explicitly indicate that the study was conducted on preschool children.

3. I believe that these qualitative preliminary studies are important for science progress. Was this qualitative study developed within a citizen science framework? Did the experimental design or questions evolve as a consequence of the interviews?

4. Were the semi-structured interviews conducted individually or in groups?

5. More information about the laypeople would help for readers understand their contribution to the study. For example, how many of the ten participants had regular contact with children? Were they parents, relatives (e.g., aunts or uncles), educators, or professionals working with children? Given their influence on the development of the project, this information appears relevant.

6. Authors described that adult creativity was often characterized as more product- and goal-oriented, and situated within specific domains such as profession or hobbies. Did the authors collect the professional background of the adults in the main study? This variable may be important, as individuals who regularly engage in creative activities have been shown to outperform the general population in creativity-related tasks (e.g., Rondini et al., “Stable Diffusion Models Reveal a Persisting Human–AI Gap in Visual Creativity”, Advanced Science, 2026). If such information was not collected, the authors should acknowledge this as a potential limitation of the study.

7. In the Introduction, the authors may wish additional constrained creative tasks involving drawing production, such as the Test of Creative Imagery Activities (TCIA; Jankowska & Karwowski, “Measuring Creative Imagery Abilities”, Frontiers in Psychology, 2015).

8. The assessment of the creative performance is a strength of the study, particularly the use of multiple raters and separate evaluations of novelty and effectiveness. However, did the authors examine the extent to which these two dimensions explain the overall creativity score? In other words, should overall creativity be interpreted as a distinct construct, or primarily as a combination of novelty and effectiveness?

9. It is not entirely clear who administered the task to the preschool children. Experimenter familiarity may influence children’s performance and should therefore be reported.

10. Why was the feasibility of the procedure initially tested by two children aged four and seven years? Since the seven year-old is no longer a preschool child, some justification for this choice would be helpful.

11. The manuscript would benefit from the inclusion of example drawings. A figure showing representative examples from the different tasks and groups would be highly informative for readers.

12. The authors indicate that the five raters were blind to participant group. However, it seems plausible that group membership could often be inferred from the drawings themselves. For example, preschool children may use fewer colors, produce less complete human figures, or display other age-related characteristics. This potential limitation should be acknowledged.

13. In Figure 2, please explicitly indicate that the measure of general creative engagement was reported by the parents.

14. Another strength of this study is the simultaneous evaluation of both the creative performance and the experience. To highlight this contribution, I suggest splitting the Figure 3 into two separate figures: one focusing on performance measures (Novelty, Effectiveness, and Creativity), and the other one on the experiential measures (Self-Efficacy, Motivation, Enjoyment, Self-Appraisal and Anxiety).

15. Considering the results across the three tasks, it is somewhat surprising that the novelty scores were lower in the free-drawing task for both groups. Could this reflect a limitation of the rating procedure, or perhaps the greater freedom of the task led participants to generate many ideas without fully developing a particularly original one? This pattern is interesting and may deserve consideration in the Discussion.

16. The description of tasks’ duration should be complemented with quantitative data. Mean durations, standard deviations, and relevant statistical analyses comparing groups should be reported within the manuscript.

**********

what does this mean?). If published, this will include your full peer review and any attached files.

If you choose “no”, your identity will remain anonymous but your review may still be made public.

**Do you want your identity to be public for this peer review?**  For information about this choice, including consent withdrawal, please see our Privacy Policy

Reviewer #2: No

Reviewer #3: **Yes:** James Russo

Reviewer #4: **Yes:** Xim Cerda-Company

**********

[NOTE: If reviewer comments were submitted as an attachment file, they will be attached to this email and accessible via the submission site. Please log into your account, locate the manuscript record, and check for the action link "View Attachments". If this link does not appear, there are no attachment files.]

To ensure your figures meet our technical requirements, please review our figure guidelines: https://journals.plos.org/plosone/s/figures

You may also use PLOS’s free figure tool, NAAS, to help you prepare publication quality figures: https://journals.plos.org/plosone/s/figures#loc-tools-for-figure-preparation.

NAAS will assess whether your figures meet our technical requirements by comparing each figure against our figure specifications.

# Author comment: Author response to Decision Letter 2

**DOI:** 10.1371/journal.pone.0346553.r004

14 Aug 2026

Point-by-Point Reviewer Response

Dear Editor, dear Reviewers,

Thank you very much for the opportunity to submit a revised version of our manuscript entitled Are children really more creative than adults? A mixed-methods examination of creative performance and experience. We are happy that the original two reviewers were already satisfied with the previous version and are grateful for the additional constructive and insightful comments provided during this review round.

Please find below a detailed description of how each Reviewer point was addressed in the revised manuscript. We believe that careful consideration of the thoughtful feedback has helped to strengthen the article substantially.

For ease of review, text changes implemented during the first revision are highlighted in yellow, while changes introduced in the present revision are highlighted in pink throughout the marked manuscript.

Sincerely,

The Authors

Reviewer #3

I found this to be a thoughtful and conceptually interesting paper that makes a worthwhile contribution to the creativity literature. In particular, I valued the authors’ attempt to disentangle creative performance from creative engagement, and to empirically examine why children are often perceived as “more creative” despite adults outperforming them on externally evaluated creative tasks. The overall pattern of findings was coherent and theoretically generative, especially the distinction between children’s stronger affective engagement (e.g., higher confidence, enjoyment, and intrinsic motivation) and adults’ stronger performance under evaluative or goal-directed conditions. I also appreciated the inclusion of lower-constraint tasks within the design, which strengthened the study by moving beyond purely structured creativity assessments. Overall, I found the paper clearly written, theoretically engaged, and addressing a genuinely interesting and culturally resonant question.

Response: We thank the reviewer for these positive and encouraging comments and are pleased that the contribution and rationale of our study were appreciated.

Additional reflections and future directions

One avenue that may be fruitful for future research concerns forms of creativity that are less product-oriented and less tightly tied to externally evaluated performance. While the inclusion of lower-constraint tasks strengthens the present design, many everyday perceptions of children’s creativity may emerge from more socially situated and play-based contexts, such as pretend play, collaborative imaginative activity, narrative improvisation, or spontaneous symbolic exploration. Investigating creativity in such settings may help further clarify the distinction observed here between creative performance and creative engagement/agency. For example, in genuinely open-ended collaborative imaginative contexts, adults may demonstrate advantages in sustaining coherent narratives, integrating abstraction or humour, and collectively scaffolding complex symbolic worlds, even while children appear more spontaneous, playful, or intrinsically immersed in the activity itself.

Response: Thank you for this valuable suggestion. We agree that future research should compare children and adults across a broader range of creative activities and consider additional indicators of creative expression beyond conventional performance measures. We have incorporated this perspective into the Limitations and future directions section:

“Fourth, other creativity tasks (e.g., insight problem-solving) and more open-ended or collaborative forms of creative activity could be included, and task order should be counterbalanced to limit fatigue effects. Future research may also consider a broader range of evaluation criteria, such as narrative coherence and collaborative idea development when comparing creativity across age groups.”

The findings also seem to create a natural opportunity for a qualitative follow-up study with the same adult participants. A particularly interesting feature of the present design is that the adult participants both completed the creativity tasks and, as a group, strongly endorsed the view that children are more creative. This places them in a unique position to help unpack the apparent disconnect between everyday beliefs about children’s creativity and the pattern of task-based performance reported in the study. For example, participants could be presented with the study’s findings and invited to reflect on whether the results surprised them, whether they viewed the administered tasks as representative of creativity more broadly, and whether their perceptions of children’s creativity are grounded more in observable engagement, spontaneity, playfulness, or imaginative expression than in externally evaluated creative products. Such a follow-up could provide particularly rich insight into some of the interpretive tensions the paper itself raises regarding the relationship between perceived creativity, creative engagement, and creative performance.

Response: Thank you for this great suggestion. We have incorporated this idea into the Future Directions section:

“Finally, as the findings are based on samples drawn from a specific cultural context, future research could examine how lay beliefs and creative expression vary across different populations and background. Yet, another avenue for future work would be to confront adults viewing children as more creative with the findings of this study and ask them to justify or elaborate their views in the light of these results.”

Reviewer #4

The study is well-structured and motivated, and it provides a valuable contribution to the creativity literature. Nevertheless, several aspects should be addressed to strengthen the manuscript and improve its overall impact.

1. As a general comment, the resolution of figures should be improved.

Response: Thank you for this comment. We re-examined all figures and confirmed that they were submitted in accordance with the journal’s requirements. We acknowledge that the figure quality is sometimes degraded in the version that is shared with the reviewers, while often providing the option to retrieve a high-resolution version via a link.

2. Based on the methods and terminology used throughout the manuscript, the title should explicitly indicate that the study was conducted on preschool children.

Response: Thank you for this suggestion. We agree that the specific age groups should be clearly communicated and have therefore clarified throughout the manuscript that the child sample consisted of preschool children. However, we chose to retain the original title because this work addressed the well-established myth of whether children are more creative than adults. The study eventually operationalized children by preschool children, because the qualitative study suggested that differences in creativity might be most pronounced in the preschool age group. For this reason, we would prefer to maintain the original title, especially after specifying the children group in all other relevant places.

3. I believe that these qualitative preliminary studies are important for science progress. Was this qualitative study developed within a citizen science framework? Did the experimental design or questions evolve as a consequence of the interviews?

Response: Thank you for this interesting question. The qualitative interviews were not conducted within a formal citizen science framework, as participants contributed solely as research participants and were not involved in the design or conduct of the research. However, the qualitative phase was central to the project and directly informed the design of the quantitative study. As described in the subsection Design implications for the main study, the interview findings guided several key design decisions, including the selection of task conditions, outcome measures, and the focus on preschool children.

4. Were the semi-structured interviews conducted individually or in groups?

Response: The interviews were conducted individually. We have clarified this in the manuscript:

“10 adult laypeople (60% female) with a mean age of 29.70 years (SD = 2.36) participated in individual semi-structured interviews designed to elicit beliefs about creativity in children and adults after giving verbal informed consent.”

5. More information about the laypeople would help for readers understand their contribution to the study. For example, how many of the ten participants had regular contact with children? Were they parents, relatives (e.g., aunts or uncles), educators, or professionals working with children? Given their influence on the development of the project, this information appears relevant.

Response: We agree that this information is relevant. Additional information on the interviewed participants, including their professional background and contact with children, is available in the study materials on the OSF. To improve transparency, we have added a reference to these materials in the manuscript:

“10 adult laypeople (60% female) with a mean age of 29.70 years (SD = 2.36) participated in individual semi-structured interviews designed to elicit beliefs about creativity in children and adults after giving verbal informed consent. Additional information on participant characteristics and study materials is available in the OSF repository.”

6. Authors described that adult creativity was often characterized as more product- and goal-oriented, and situated within specific domains such as profession or hobbies. Did the authors collect the professional background of the adults in the main study? This variable may be important, as individuals who regularly engage in creative activities have been shown to outperform the general population in creativity-related tasks (e.g., Rondini et al., “Stable Diffusion Models Reveal a Persisting Human–AI Gap in Visual Creativity”, Advanced Science, 2026). If such information was not collected, the authors should acknowledge this as a potential limitation of the study.

Response: Thank you for this comment. We did not collect detailed information on participants’ professional background or engagement in creative professions. While the adult sample was recruited from the general population and was not specifically selected based on creative expertise, we agree that individual difference in creative expression may influence performance on creativity tasks. We have therefore acknowledged this point as a limitation in the revised manuscript:

“Fifth, creative products were rated by adults; children might evaluate children’s products differently. In addition, we did not assess participants’ professional background or creative expertise, which may represent an additional source of variability in creative performance.”

7. In the Introduction, the authors may wish additional constrained creative tasks involving drawing production, such as the Test of Creative Imagery Activities (TCIA; Jankowska & Karwowski, “Measuring Creative Imagery Abilities”, Frontiers in Psychology, 2015).

Response: Thank you for this suggestion. We have added the TCIA as an additional example in the introduction.

8. The assessment of the creative performance is a strength of the study, particularly the use of multiple raters and separate evaluations of novelty and effectiveness. However, did the authors examine the extent to which these two dimensions explain the overall creativity score? In other words, should overall creativity be interpreted as a distinct construct, or primarily as a combination of novelty and effectiveness?

Response: Thank you for this insightful comment. To further examine the relationship between novelty, effectiveness, and overall creativity, we calculated response-level correlations for each task and report them in the Supplementary Materials (see Table S2). We also added a reference to these analyses in the Scoring of creative performance section. Overall creativity was strongly associated with novelty across all tasks, whereas associations with effectiveness varied substantially by task. These findings suggest that overall creativity judgments were closely linked to novelty, but that effectiveness ratings offered a complementary view on creative quality especially in the AUT.

“Interrater reliability was high across tasks and dimensions, with Cronbach’s α ranging from .789 to .980. Full reliability coefficients, including 95% confidence intervals, are reported in the Supplemental Analyses (see Table S1). Response-level correlations among novelty, effectiveness, and overall creativity ratings replicated that creativity evaluations are more strongly driven by the perceived novelty than effectiveness; detailed analyses are reported in the Supplemental Analyses (see Table S2).”

S2 Table. Response-level correlations among novelty, effectiveness, and overall creativity ratings across creativity tasks. * p < .05. ** p < .01. *** p < .001.

Task r(Novelty, Effectiveness) r (Novelty, Creativity) r (Effectiveness, Creativity)

Alternate Uses Task -.09* .92*** .12**

Structured Creative Drawing .90*** .96*** .96***

Free Drawing .39*** .78*** .83***

9. It is not entirely clear who administered the task to the preschool children. Experimenter familiarity may influence children’s performance and should therefore be reported.

Response: All participants were assessed individually by one of the authors, who was not familiar with the participants prior to testing. We have clarified this in the manuscript:

“All participants were tested individually between August 1, 2021, and April 30, 2022, by one of the authors, who was not familiar with the participants prior to testing.”

10. Why was the feasibility of the procedure initially tested by two children aged four and seven years? Since the seven year-old is no longer a preschool child, some justification for this choice would be helpful.

Response: The pilot testing was conducted to evaluate the comprehensibility and feasibility of the procedure and task instructions before the main study. At this stage, children from a somewhat broader age range were included to ensure that the tasks could be understood and completed appropriately across different developmental levels. We have clarified this rationale in the manuscript:

“The procedure was initially pilot-tested with two children (four and seven years old) to evaluate the comprehensibility and feasibility of the tasks and instructions across different developmental levels; the results informed final task adjustments.”

11. The manuscript would benefit from the inclusion of example drawings. A figure showing representative examples from the different tasks and groups would be highly informative for readers.

Response: We agree that example drawings can help readers better understand the nature of the drawing tasks and the types of responses obtained. While publication permission was not available for all participants, a subset of participants explicitly consented to the use of their drawings for publication purposes. We therefore added a selection of representative example drawings from these participants to the online supplementary materials and included a reference to these materials in the manuscript.

12. The authors indicate that the five raters were blind to participant group. However, it seems plausible that group membership could often be inferred from the drawings themselves. For example, preschool children may use fewer colors, produce less complete human figures, or display other age-related characteristics. This potential limitation should be acknowledged.

Response: Thank you for this important observation. Although raters were not informed about participants’ age group, we agree that age-related characteristics of the drawings may have allowed group membership to be inferred in some cases. We have acknowledged this as a limitation in the revised manuscript:

“In addition, we did not assess participants’ professional background or creative expertise, which may represent an additional source of variability in creative performance. Moreover, although raters were blind to participants’ age group, age-related characteristics of the drawings may have allowed group membership to be inferred in some cases.”

13. In Figure 2, please explicitly indicate that the measure of general creative engagement was reported by the parents.

Response: We have clarified this in the figure note:

“Fig 2. General creative engagement, creative self-concept, and beliefs about creativity. For preschool children, ratings of general creative engagement were provided by parents, whereas all other measures were self-reported.”

14. Another strength of this study is the simultaneous evaluation of both the creative performance and the experience. To highlight this contribution, I suggest splitting the Figure 3 into two separate figures: one focusing on performance measures (Novelty, Effectiveness, and Creativity), and the other one on the experiential measures (Self-Efficacy, Motivation, Enjoyment, Self-Appraisal and Anxiety).

Response: Thank you for this helpful suggestion. To better distinguish between creative performance and creative experience, we reorganized Figure 3 and added separate section headings for both domains.

15. Considering the results across the three tasks, it is somewhat surprising that the novelty scores were lower in the free-drawing task for both groups. Could this reflect a limitation of the rating procedure, or perhaps the greater freedom of the task led participants to generate many ideas without fully developing a particularly original one? This pattern is interesting and may deserve consideration in the Discussion.

Response: Thank you for this interesting observation. We agree that the pattern deserves consideration. We believe that the lower novelty ratings in the free-drawing task are likely attributable to the task instructions. Whereas the structured drawing task explicitly encouraged participants to create a novel fantasy animal, the free-drawing task allowed participants to draw any subject, including highly conventional and familiar objects. We have clarified this point in the manuscript:

“Adults showed consistently higher task performance than the preschool children in the AUT and the structured drawing task, as their ideas and drawings were rated as more novel, effective, and creative. In the free drawing task, adults also produced more effective and creative drawings than children, but groups did not differ in terms of novelty as adults often chose similarly common drawing topics. This pattern is consistent with the task instructions, as only the structured drawing task explicitly encouraged the production of a creative response.”

16. The description of tasks’ duration should be complemented with quantitative data. Mean durations, standard deviations, and relevant statistical analyses comparing groups should be reported within the manuscript.

Response: We agree that the description of task duration should be accompanied by quantitative information. We have therefore added the corresponding descriptive statistics to the manuscript:

“Interestingly, children spent less time than adults on the timed AUT [M = 1.92, SD = 0.87 vs. M = 2.72, SD = 0.61; t(159) = -6.46, p < .001] and the structured drawing task [M = 2.71, SD = 2.32 vs. M = 6.58, SD = 1.92; t(159) = -11.63, p < .001]; in the unstructured drawing task, which was untimed, children (M = 5.06, SD = 4.66) and adults (M = 4.16, SD = 2.90) spent a similar amount of time [t(154) = 1.50, p = .134].”

Attachment Submitted filename: Response_to_Reviewers.docx

# Peer review: Decision Letter 2

**DOI:** 10.1371/journal.pone.0346553.r005

**Authors:** Mohammad Salah Hassan

14 Sep 2026

<p>Are children really more creative than adults?

A mixed-methods examination of creative performance and experience

PONE-D-26-13778R2

Dear Dr. Authors,

We’re pleased to inform you that your manuscript has been judged scientifically suitable for publication and will be formally accepted for publication once it meets all outstanding technical requirements.

Within one week, you’ll receive an e-mail detailing the required amendments. When these have been addressed, you’ll receive a formal acceptance letter and your manuscript will be scheduled for publication.

An invoice will be generated when your article is formally accepted. Please note, if your institution has a publishing partnership with PLOS and your article meets the relevant criteria, all or part of your publication costs will be covered. Please make sure your user information is up-to-date by logging into Editorial Manager at Editorial Manager® and clicking the ‘Update My Information' link at the top of the page. For questions related to billing, please contact billing support.

If your institution or institutions have a press office, please notify them about your upcoming paper to help maximize its impact. If they’ll be preparing press materials, please inform our press team as soon as possible -- no later than 48 hours after receiving the formal acceptance. Your manuscript will remain under strict press embargo until 2 pm Eastern Time on the date of publication. For more information, please contact onepress@plos.org.

Kind regards,

Mohammad Salah Hassan, Ph.D

Academic Editor

PLOS One

Additional Editor Comments (optional):

Dear Authors,

Thank you for carefully addressing the reviewers’ comments and revising the manuscript accordingly. The manuscript has improved substantially, and the reviewers are satisfied with the revisions.

I am pleased to recommend the manuscript for acceptance.

Best regards,

Reviewers' comments:

Reviewer's Responses to Questions

**Comments to the Author**

Reviewer #4: All comments have been addressed

**********

2. Is the manuscript technically sound, and do the data support the conclusions?

Reviewer #4: Yes

**********

3. Has the statistical analysis been performed appropriately and rigorously?

Reviewer #4: Yes

**********

4. Have the authors made all data underlying the findings in their manuscript fully available?

The PLOS Data policy

Reviewer #4: Yes

**********

5. Is the manuscript presented in an intelligible fashion and written in standard English?

Reviewer #4: Yes

**********

Reviewer #4: Although the authors have included some modifications in the Supplementary Materials (e.g., creativity score), all comments have been addressed.

**********

what does this mean?). If published, this will include your full peer review and any attached files.

If you choose “no”, your identity will remain anonymous but your review may still be made public.

**Do you want your identity to be public for this peer review?**  For information about this choice, including consent withdrawal, please see our Privacy Policy

Reviewer #4: **Yes:** Xim Cerda-Company

**********

# Editor report: Acceptance letter

**DOI:** 10.1371/journal.pone.0346553.r006

**Authors:** Mohammad Salah Hassan

PONE-D-26-13778R2

PLOS One

Dear Dr. Benedek,

I'm pleased to inform you that your manuscript has been deemed suitable for publication in PLOS One. Congratulations! Your manuscript is now being handed over to our production team.

At this stage, our production department will prepare your paper for publication. This includes ensuring the following:

* All references, tables, and figures are properly cited

* All relevant supporting information is included in the manuscript submission,

* There are no issues that prevent the paper from being properly typeset

You will receive further instructions from the production team, including instructions on how to review your proof when it is ready. Please keep in mind that we are working through a large volume of accepted articles, so please give us a few days to review your paper and let you know the next and final steps.

Lastly, if your institution or institutions have a press office, please let them know about your upcoming paper now to help maximize its impact. If they'll be preparing press materials, please inform our press team within the next 48 hours. Your manuscript will remain under strict press embargo until 2 pm Eastern Time on the date of publication. For more information, please contact onepress@plos.org.

You will receive an invoice from PLOS for your publication fee after your manuscript has reached the completed accept phase. If you receive an email requesting payment before acceptance or for any other service, this may be a phishing scheme. Learn how to identify phishing emails and protect your accounts at https://explore.plos.org/phishing.

If we can help with anything else, please email us at customercare@plos.org.

Thank you for submitting your work to PLOS One and supporting open access.

Kind regards,

PLOS One Editorial Office Staff

on behalf of

Dr. Mohammad Salah Hassan

Academic Editor

PLOS One
